# Nutrition-induced changes in the microbiota can cause dysbiosis and disease development

**DOI:** 10.1128/mbio.03843-24

**Published:** 2025-02-25

**Authors:** Tim Lachnit, Laura Ulrich, Fiete M. Willmer, Tim Hasenbein, Leon X. Steiner, Maria Wolters, Eva M. Herbst, Peter Deines

**Affiliations:** 1 Zoological Institute, Christian-Albrechts-University Kiel, Kiel, Germany; 2 Institute of Human Genetics, University Hospital Schleswig-Holstein, Kiel, Germany; 3 Institute of Pharmacology and Toxicology, Technical University of Munich, München, Germany; 4 RU Marine Symbioses, RD3 Marine Ecology, GEOMAR Helmholtz Centre for Ocean Research, Kiel, Germany; 5 Fakultät Nachhaltigkeit, Leuphana Universität Lüneburg, Lüneburg, Germany; 6 Experimental Orthopedics and Trauma Surgery, University Hospital Schleswig-Holstein, Kiel, Germany; University of Hawaii at Manoa, Honolulu, Hawaii, USA

**Keywords:** microbiota, microbiome, dysbiosis, disease, pathogens, nutrition, inflammation, host-microbe interaction, environment

## Abstract

Eukaryotic organisms are associated with complex microbial communities. Changes within these communities have been implicated in disease development. Nonetheless, it remains unclear whether these changes are a cause or a consequence of disease. Here, we report a causal link between environment-induced shifts in the microbiota and disease development. Using the model organism *Hydra*, we observed changes in microbial composition when transferring laboratory-grown *Hydra* to natural lake environments. These shifts were caused not only by new colonizers, through the process of community coalescence (merging of previously separate microbial communities), but also by lake water nutrients. Moreover, selective manipulation of the nutrient environment induced compound-specific shifts in the microbiota followed by disease development. Finally, L-arginine supplementation alone caused a transition in *Pseudomonas* from symbiotic to pathogenic, leading to an upregulation of immune response genes, tissue degradation, and host death. These findings challenge the notion that the host-associated microbiota is exclusively controlled by the host, highlighting the dynamic interplay between host epithelial environment, microbial colonizer pool, and nutrient conditions of the surrounding water. Furthermore, our results show that overfeeding of the microbiota allows for uncontrolled microbial growth and versatile interactions with the host. Environmental conditions may thus render symbionts a potential hazard to their hosts, blurring the divide between pathogenic and non-pathogenic microbes.

**IMPORTANCE:**

This study highlights the critical need to understand the dynamic interplay between host-associated microbiota and environmental factors to obtain a holistic view on organismal health. Our results demonstrate that ecosystem-wide microbial trafficking (community coalescence) and environmental nutrient conditions reshape microbial communities with profound implications for host health. By exploring nutrient-driven changes in microbial composition, our research finds experimental support for the “overfeeding hypothesis,” which states that overfeeding alters the functionality of the host microbiota such that an overabundance in nutrients can facilitate disease development, transforming non-pathogenic microbes into pathogens. These findings emphasize the critical role of metabolic interactions driving microbial pathogenicity. Furthermore, our research provides empirical evidence for the “pathogenic potential” concept, challenging traditional distinctions between pathogenic and non-pathogenic microbes and supporting the idea that any microbe can become pathogenic under certain conditions.

## INTRODUCTION

The prevalence of inflammatory and autoimmune diseases is rising at an alarming rate on a global scale. Despite the advancements in therapeutic approaches, our understanding of the underlying causes remains limited. One way to improve our understanding of the origins of these complex diseases is to consider that all eukaryotic organisms, including plants, animals, and humans, are associated with complex microbial communities (1, 2). This microbiota frequently affects host’s health, behavior, and, under certain conditions, fitness-determining traits of the host, including growth rate, reproduction, survival, and aging (3). Microbial community analysis offers insight into the origin of complex diseases, such as the hypothesis that compositional and functional alterations in host-associated microbial communities (dysbiosis) may be the causal factor in disease development. For instance, several human diseases, including inflammatory bowel disease (4), multiple sclerosis (5), atopic dermatitis (6), and type 2 diabetes (7), are associated with alterations in microbial community composition. While a causal link between changes in the microbiota and disease etiology has not yet been established, genetic predisposition presents another contributing factor in the development and manifestation of complex diseases (8–10). However, inherited genetic risk factors for complex diseases can only account for a relatively small percentage of disease development, while environmental factors are considered to be the primary trigger for disease onset (11). It is assumed that the post-modern industrial lifestyle, characterized by excess nutrient intake and the consumption of high-calorie processed foods, has the potential to decouple natural host-microbe associations. Such changes result in functional alterations, increased growth of microbes, higher concentrations of microbial by-products, and shifts in microbial community composition, and ultimately facilitate the development of disease, as proposed by the “overfeeding hypothesis” (12). This effect is amplified by the loss of important microbes through the use of antibiotics and the limited availability of new bacterial colonizers due to increased sanitation, as outlined in the “hygiene hypothesis” (13). Such a loss in microbial diversity may negatively affect the resilience of the microbiota and the capacity of hosts to adapt to environmental changes. In combination, these hypotheses hint at the complex interplay between nutrients, the natural environment with its microbes, and the microbiome of organisms.

The structure and diversity of host-associated microbial communities are determined by complex and dynamic interactions between the host genotype and the microbial population’s genetic diversity (14), host diet (15), and the local environment (16). Features of the local environment, including water temperature, salinity, pH, and nutrient availability, can affect diversity and structure of host microbiomes. Nevertheless, the significance of these factors is only beginning to emerge, while the mechanisms underlying variation in microbial diversity and disease emergence remain poorly understood.

It was shown that the composition of the microbiome of the freshwater cnidarian *Hydra* is actively shaped by the host through the secretion of antimicrobial peptides (17, 18). Moreover, the role of its microbiome in developmental processes, tissue homeostasis, physiological and behavioral performance, and protection against fungal infections is well-documented (19–22). Early studies have shown that *Hydra* species differ in their microbiota and that species-specific microbiota persist long term (17, 23). *Hydra*’s ectodermal epithelial cells are covered with a multilayered glycocalyx that serves as a habitat for its microbiota (12, 24). As an aquatic organism, *Hydra*’s microbiome is in direct contact with the surrounding water, both with its microbes and nutrients. Such an interchange between entire communities has recently been termed community coalescence (25, 26) and represents an underexplored phenomenon in host microbiome ecology.

We employed a multifaceted approach, combining observational field studies with controlled laboratory experiments, in order to reveal how ecosystems can generate selective pressures that result in the emergence of a pathobiont (a microorganism that can cause harm under certain conditions). To uncover ecological drivers leading to the selection of pathogenic traits, we conducted nutrient manipulation experiments on wild-type (wt) and mono-colonized organisms, to establish causality and explore underlying mechanisms. Our first aim was to disentangle the contribution of environmental microbes and nutrients on changes in host-associated microbial communities. The relocation of laboratory *Hydra* to lakes with varying nutrient concentrations permitted us to observe changes in their microbiome over time. Further laboratory experiments confirmed that exposing polyps to diverse nutrient environments induced a disease phenotype but only in the presence of microbiota. Germ-free polyps remained unaffected. The disease phenotype correlated with an increase of a *Pseudomonas* sp. and the presence of specific nutrients. After isolating this strain, we studied the host’s transcriptomic responses as well as the transcriptome of this particular *Pseudomonas* sp. during disease development. Our study shows that the outcome of host-microbe interactions is inherently unpredictable and that increased levels of one amino acid transform a symbiont into a lethal pathobiont.

## RESULTS

### The environment has a strong impact on microbial community composition

**Fig 1:**
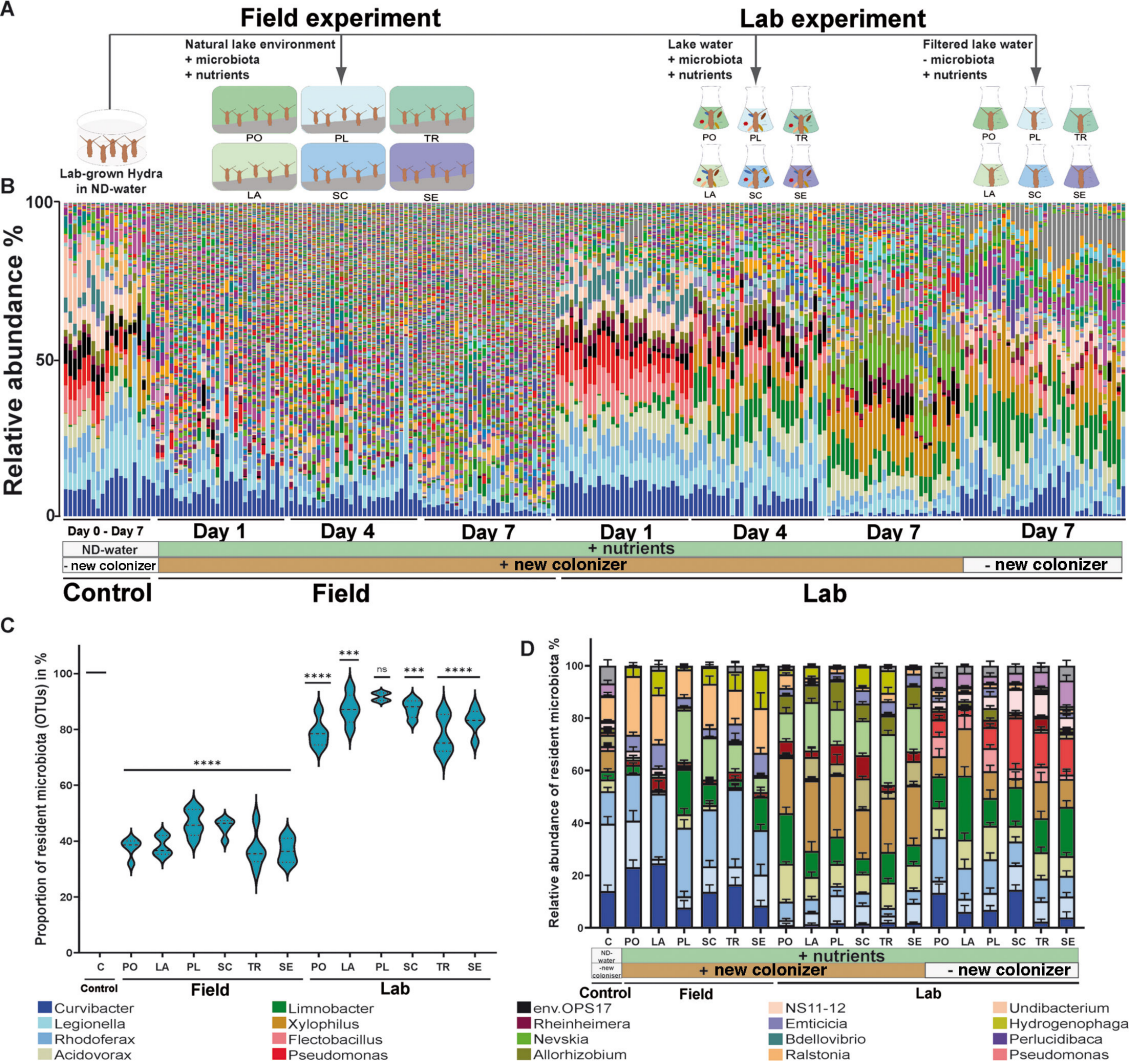
Natural lake environments have a strong impact on *Hydra*’s microbial community composition. (**A**) Graphical illustration of the experimental design. Laboratory-grown *Hydra vulgaris* (AEP) polyps in nutrient-deficient water (ND-water) were transferred to different lake environments in the field or to the corresponding lake water under laboratory conditions. The abbreviations PO, LA, PL, SC, TR, and SE denote specific lakes, with full names and geographical locations provided in Fig. S1. In addition, laboratory-grown *Hydra* polyps were exposed to sterile, filtered lake water in the absence of new microbes. (**B**) Bar chart illustrating microbial community composition based on operational taxonomic units (OTUs). (**C**) Proportion of resident bacterial OTUs compared to new colonizers. Dotted lines in violin plots represent the median. One-way analysis of variance with Dunnett’s multiple comparison test compares each group with the control (*P* > 0.01 = not significant [ns]; ***P < 0.001; ****P < 0.0001). (**D**) Environmental changes affect the resident core microbial communities. Resident bacterial OTUs undergo abundance shifts in the field and when exposed to lake water in the laboratory. Exposing *Hydra* only to sterile, filtered lake water in the absence of any microbes already had an impact on microbial community composition. Data are mean ± SE.

The aim of our study was to investigate *Hydra*’s ability to maintain its host-specific microbial community composition under different environmental conditions. To achieve this, laboratory-grown polyps were relocated to natural lakes with different nutrient levels (Fig. S1) and simultaneously exposed to lake water under controlled laboratory conditions (Fig. 1A). Both microbial community compositions were monitored over time.

For up to 7 days, exposure of laboratory-grown *Hydra* to different lake environments in the field and under controlled laboratory conditions had a significant effect on the composition of the host-associated microbial community (Fig. S2). 16S rRNA gene sequencing analysis revealed that the associated microbial community of *Hydra* had undergone a notable change in polyps exposed to lake water under laboratory conditions. This effect was even more pronounced in *Hydra* polyps exposed to natural field conditions (Fig. 1B).

The present study focused its analysis on the resident microbiota, defined as the bacterial species identified within the *Hydra* microbiota prior to its exposure to the lake environment. Over time, the relative abundance of *Hydra*-specific bacteria decreased significantly. Starting from an initial abundance of 100%, the total relative abundance under laboratory culture conditions declined to approximately 80% after 7 days. In the field, the relative abundance decreased to approximately 40% after 7 days (Fig. 1C). Consequently, the relative abundance of resident microbes was higher when polyps were exposed to lake water under laboratory conditions. It can be concluded that the changes in relative abundance were not solely caused by the additional colonization of bacteria from the surrounding lake water. By computationally excluding all new microbial colonizers and normalizing the resident microbiota to a relative abundance of 100%, we demonstrated that the resident microbiota itself underwent compositional shifts in the lake environment (Fig. 1D). Specifically, the abundance of *Curvibacter* and *Legionella* decreased, while the relative abundance of other bacteria exhibited fluctuations (e.g., *Rhodoferax*). In contrast, microbes that were normally underrepresented under laboratory culture conditions (e.g., *Limnobacter* and *Nevskia*) increased manifold in abundance (Fig. 1D).

### The chemical environment induces shifts in the resident microbiota

To test whether microbial community shifts were facilitated by invading microbes from the water column or caused by the altered chemical environment, we removed microbes through filtration and exposed *Hydra* solely to the water chemistry of the different lake environments under laboratory conditions. As a control, *Hydra* was cultured in nutrient-deficient water (27), which lacks natural nutrient sources for bacteria typically found in lake environments, such as dissolved organic matter and particulate organic matter.

Our findings demonstrate that the resident microbiota’s composition was affected by the lake environment’s differing water chemistry, even in the absence of potential new microbial colonizers (Fig. 1D). In particular, nutrients within the lake water had a significant impact on the composition of the microbial community. More specifically, we observed a decrease in *Curvibacter* and *Legionella*, while *Acidovorax*, *Xylophilus*, *Limnobacter*, and *Pseudomonas* showed an increase in abundance compared to the control in nutrient-deficient water (Fig. 1D). It is noteworthy that our findings revealed not only differences between lake water and nutrient-deficient water but also variations in microbial community composition, depending on which lake water they were exposed to. This variation can partly be explained by differences in dissolved organic carbon (DOC) and phosphate concentrations. By analyzing the nutrient load and composition of the different lake waters, including measurements of DOC, total phosphate, and total nitrogen (Table S3), we found that, e.g., *Acidovorax* and *Xylophilus* exhibited a positive correlation with increasing DOC levels (Fig. S3), whereas *Rhodoferax* and *Flectobacillus* demonstrated a positive correlation with the total amount of phosphate (Fig. S4). Furthermore, it was observed that the influence of nutrients on the microbiota not only was limited to quantity but also extended to nutrient composition. The ratio of DOC to nitrogen or phosphate was calculated, which revealed that decreasing C:N ratios were positively correlated with the relative abundance of *Pseudomonas*, and decreasing C:P ratios were positively correlated with *Curvibacter* (Fig. S5).

### Elevation of nutrient levels induces dysbiosis and disease development

**Fig 2:**
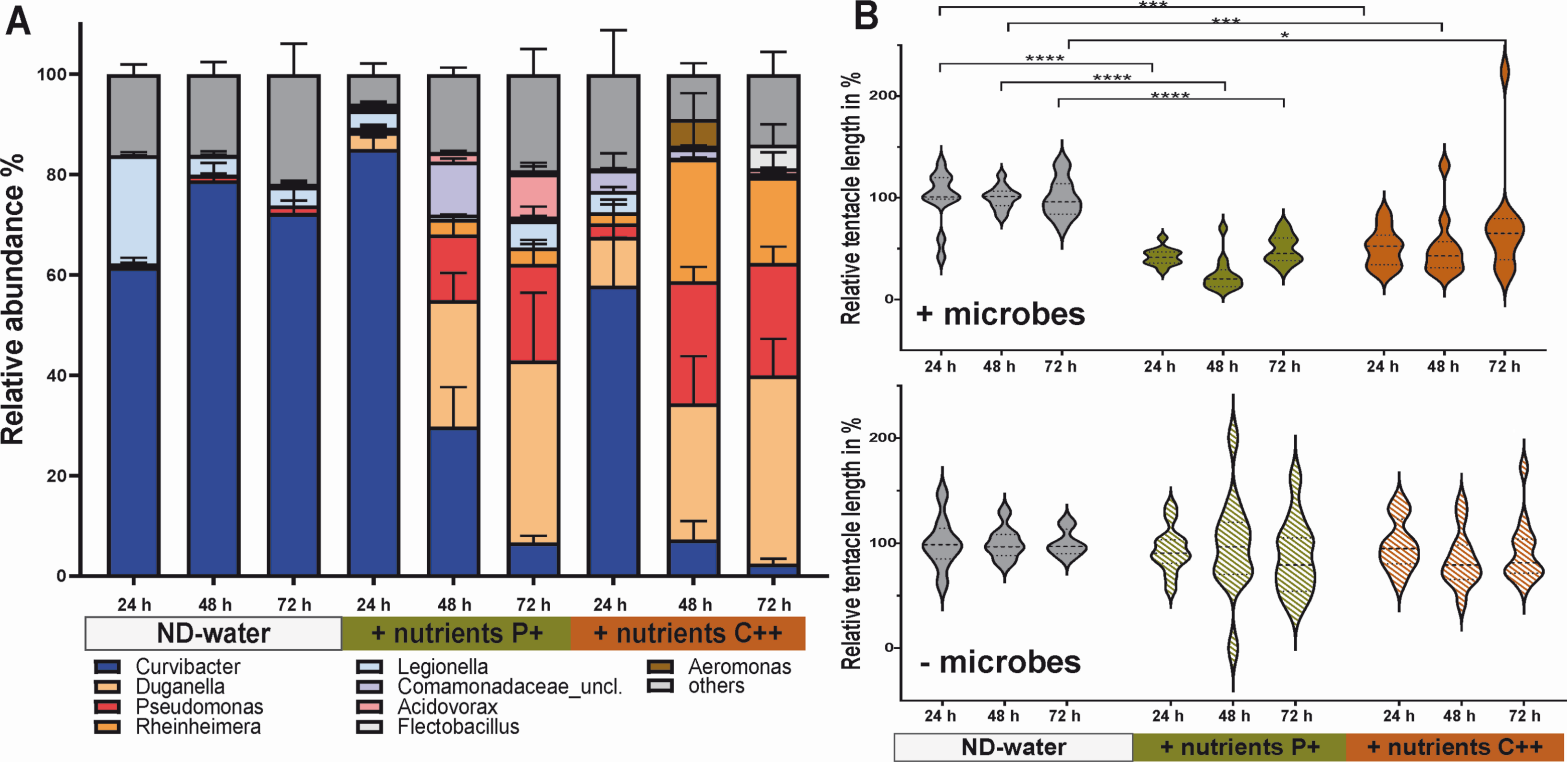
Elevated nutrient levels induce dysbiosis and disease development. (**A**) Laboratory-grown *Hydra vulgaris* (AEP) polyps were exposed to different nutrient environments, including nutrient-deficient water (ND-water) as control, a protein source (P+) and a complex nutrient source (C++). Bar chart illustrating shifts in microbial community composition based on OTUs following exposure to P+ and C++ and nutrient-deficient water as control. (**B**) Measurement of polyp tentacle length as an indicator of disease development (+microbes). Dotted lines in violin plots represent the median. One-way analysis of variance with Dunnett’s multiple comparison test compares each group with the control (*P* > 0.05 = not significant; *P < 0.01; ***P < 0.0001; ****P < 0.00001). Germ-free polyps (−microbes) were used as a control for direct effects of nutrient enrichment in the absence of microbiota.

To further elucidate the impact of nutrients on the host-associated microbiota, we exposed laboratory-grown *Hydra* to two distinct nutrient regimes (P+ and C++) in the absence of new microbial colonizers. Artificial enrichment of the *Hydra* medium (nutrient-deficient water) with nutrients resulted in a modification of the microbial community composition (Fig. S6). In both nutrient-enriched environments, the relative abundance of *Curvibacter* significantly decreased, dropping from 60%–80% (control) to less than 7% (Fig. 2A). Concurrent with the collapse of *Curvibacter*, an increase was observed in previously underrepresented *Pseudomonas*, *Duganella*, and *Rheinheimera* (Fig. 2A).

Most intriguingly, *Hydra* developed a disease phenotype with shortened tentacles (28, 29) under both nutrient-enriched environmental conditions. When polyps were exposed to enriched conditions, tentacle length was reduced by 50% within 24 h and remained significantly shorter, compared to control polyps, until the end of the experiment (Fig. 2B). This disease phenotype was observed only when polyps were associated with their microbiome. Tentacle length in germ-free (GF) polyps did not change significantly in nutrient-enriched environments compared to the control (Fig. 2B).

**Fig 3:**
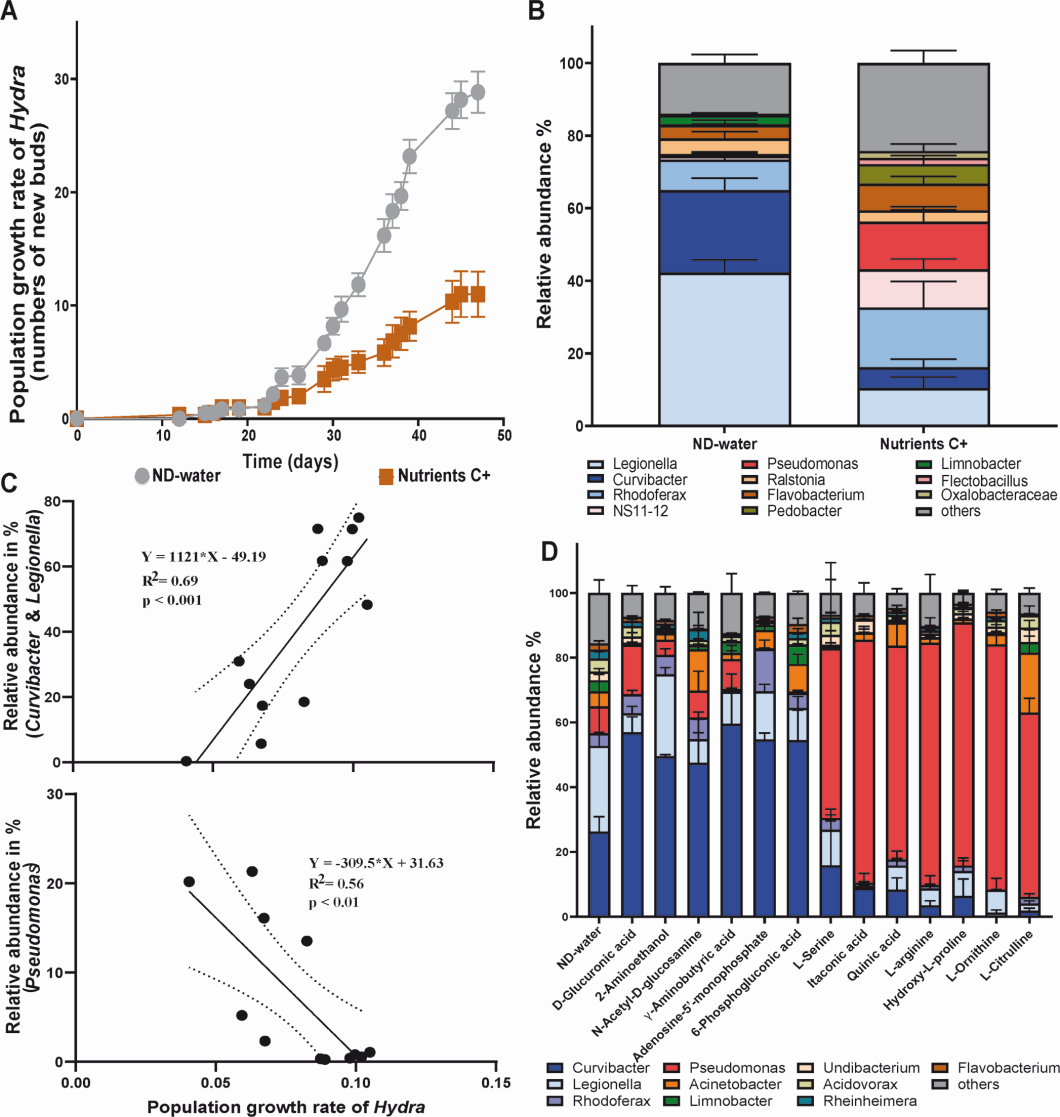
(**A**) Population growth experiment of *Hydra* exposed to nutrient-deficient water (ND-water) or a nutrient-enriched environment (C+) over a period of 47 days. Population growth was measured by counting bud production per individual polyp (*n* = 6). (**B**) Microbial community composition of the population growth experiment analyzed after 47 days of exposure to ND-water or to nutrient-enriched (C+) environment. (**C**) *Hydra* population growth rates correlate positively with the relative abundance of *Curvibacter* and *Legionella* (top) and negatively with increasing relative abundance of *Pseudomonas* (bottom). (**D**) Individual polyps were also exposed to different compounds in Biolog plates with replication (*n* = 3), allowing analysis of compound-specific effects on microbial community composition. Bar charts illustrating compound-specific shifts in relative abundance of *Hydra* microbiota after 48 h inoculation, highlighting the impact of individual compounds on the microbial community with a specific focus on *Curvibacter* and *Pseudomonas*. Data are mean ± SE.

The P+ regime (protein source) induced similar shifts in microbial community composition as the C++ regime (complex nutrient source), leading to disease development at a lower concentration. To establish whether this was a general phenomenon, the nutrient concentration was reduced to 30 mg/L (C+), which corresponds to the dissolved organic load of eutrophic lake environments (30), in a secondary experiment. This concentration did not induce visible disease symptoms but negatively affected the population growth of *Hydra* (Fig. 3A). Over a period of 47 days, polyps exposed to slightly elevated C+ concentrations produced an average of 12 buds per polyp, in comparison to 32 buds produced by polyps not exposed to nutrients. This effect of elevated nutrients on population growth was accompanied by changes in the microbial community (Fig. 3B). A microbial community analysis conducted at the end of the experiment indicated that the population growth rate of *Hydra* was positively correlated with the abundance of *Curvibacter* and *Legionella*, while slowed population growth was associated with an increasing *Pseudomonas* abundance (Fig. 3C).

### Compound-specific shifts in the microbial community composition

Our results so far demonstrated two key patterns in the relationship between nutrients and the microbiota. Firstly, the nutrient load and composition of lake water, in particular the carbon-to-nitrogen (C:N) or carbon-to-phosphate (C:P) ratio, significantly influenced the microbial community composition of the host. Secondly, the artificial enrichment of water with a protein or complex nutrient source affected *Hydra*, often to the polyp’s detriment, based on the concentration of the added nutrient. These observations strongly suggest that external feeding of the microbiota plays a pivotal role in the development of dysbiosis and disease. Furthermore, the onset of disease and reduction in population growth were both linked to an increased abundance of *Pseudomonas* and a decline in *Curvibacter*.

In order to gain insight into the impact of individual compounds on the microbiota’s composition and to identify substances that selectively promote *Pseudomonas* growth, we exposed *Hydra* polyps to a range of different substances in Biolog plates. After characterizing the composition of the *Hydra*-associated microbial community after 48 h, we detected considerable shifts within the microbial community in response to the presence of specific compounds (Fig. S7 to S10).

These shifts revealed two distinct community states: one dominated by *Curvibacter* and the other by *Pseudomonas* (Fig. S7 and S10). *Curvibacter*, one of *Hydra*’s most abundant colonizers under laboratory conditions, showed pronounced fluctuations in abundance. For instance, in the presence of γ-aminobutyric acid, *Curvibacter* attained a relative abundance of 60% and declined to less than 5% in L-citruline (Fig. 3D).

*Pseudomonas*, which typically constitutes less than 10% of the microbiota in nutrient-deficient water, demonstrated a pronounced increase to 74% when *Hydra* was exposed to the amino acid L-arginine (Fig. 3D). A similar trend was observed for few other substrates.

### Harmful infection that originates within the body

#### Changing nutrient-environment conditions turn a symbiont into a pathobiont

**Fig 4:**
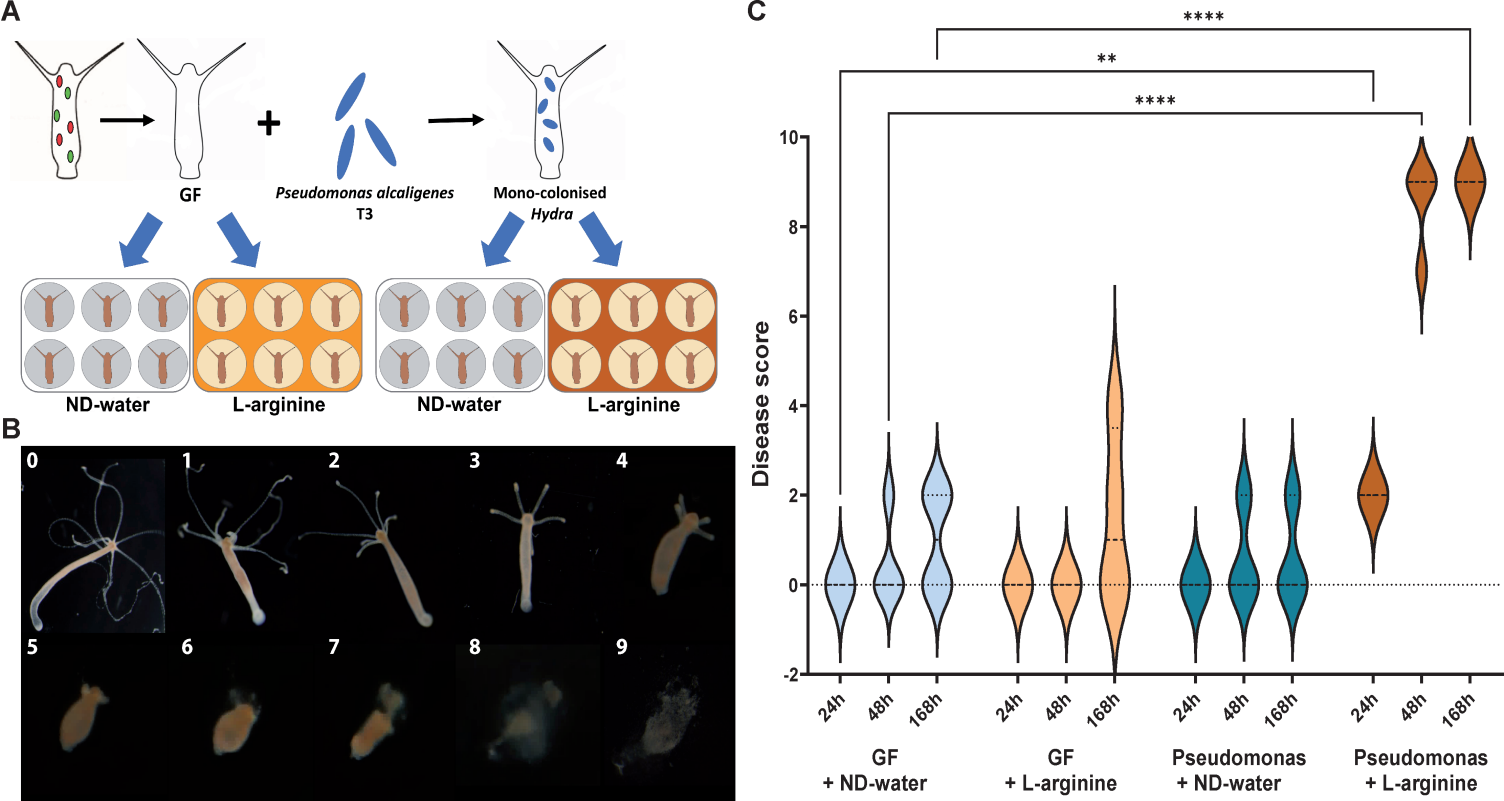
Increased nutrient levels of L-arginine turn a symbiont into a lethal pathobiont. (**A**) Experimental design: germ-free (GF) and mono-colonized polyps were exposed to either nutrient-deficient water (ND-water) or L-arginine, with replication (*n* = 6). (**B**) Disease development in mono-colonized polyps exposed to L-arginine. Disease severity was assessed by grading symptoms. Severity was determined using a numerical scale, starting with a score of 0 for healthy polyps. Reduction in tentacle length and eventual lysis of polyp tissue resulted in scores of 4–9. (**C**) Disease states of polyps were characterized by a scoring system following exposure to either ND-water or L-arginine. GF polyps exposed to nutrient-deficient water (light blue), GF polyps exposed to L-arginine (light brown), mono-colonized polyps exposed to nutrient-deficient water (dark blue), and mono-colonized polyps exposed to L-arginine (brown). Dotted lines in violin plots represent the median. One-way analysis of variance with Dunnett’s multiple comparison test compares each group with the control (**P < 0.001; ****P < 0.00001).

In all our experiments, we observed that disease development and reduced population growth were associated with an increase in the relative abundance of *Pseudomonas*. In order to gain a deeper understanding of the underlying mechanisms, we further reduced the complexity of our system. In light of our previous observation that the relative abundance of *Pseudomonas* increased in the presence of L-arginine (Fig. 3D), we mono-colonized polyps with *Pseudomonas alcaligenes* T3 (Fig. S11), an isolate from diseased polyps, and exposed them to L-arginine (Fig. 4A). Under these conditions, *Hydra* developed a pronounced disease phenotype. Disease progression was characterized by initial tentacle shrinkage and agglomeration, as well as body length reduction within the first 24 h of exposure. Tentacles then began to disintegrate and polyps finally dissolved within 48 h. Mono-colonized polyps exposed to nutrient-deficient water and GF polyps exposed to nutrient-deficient water or L-arginine did not develop the disease phenotype (Fig. 4B and C).

Contrary to our expectations, *Pseudomonas* abundance did not increase significantly during disease progression. Throughout the shrinkage process and until *Hydra*’s tentacles began to disintegrate, CFUs per polyp remained constant (Fig. S12). As soon as the tentacles began to disintegrate, CFUs increased 100-fold (Fig. S12).

In order to gain deeper insight into regulatory properties and disease-related transcriptional changes, transcriptomic analysis was conducted on *Hydra* at an early stage of disease development and 24 h post-exposure. RNA sequencing revealed that *P. alcaligenes* T3 colonization in nutrient-deficient water only had minor effects on transcription levels in *Hydra* compared to GF polyps (Fig. S13), since only 39 genes were differentially expressed (Fig. S13 and S14).

#### L-arginine induced transition of *Pseudomonas* from a non-pathogenic to a pathogenic state

**Fig 5:**
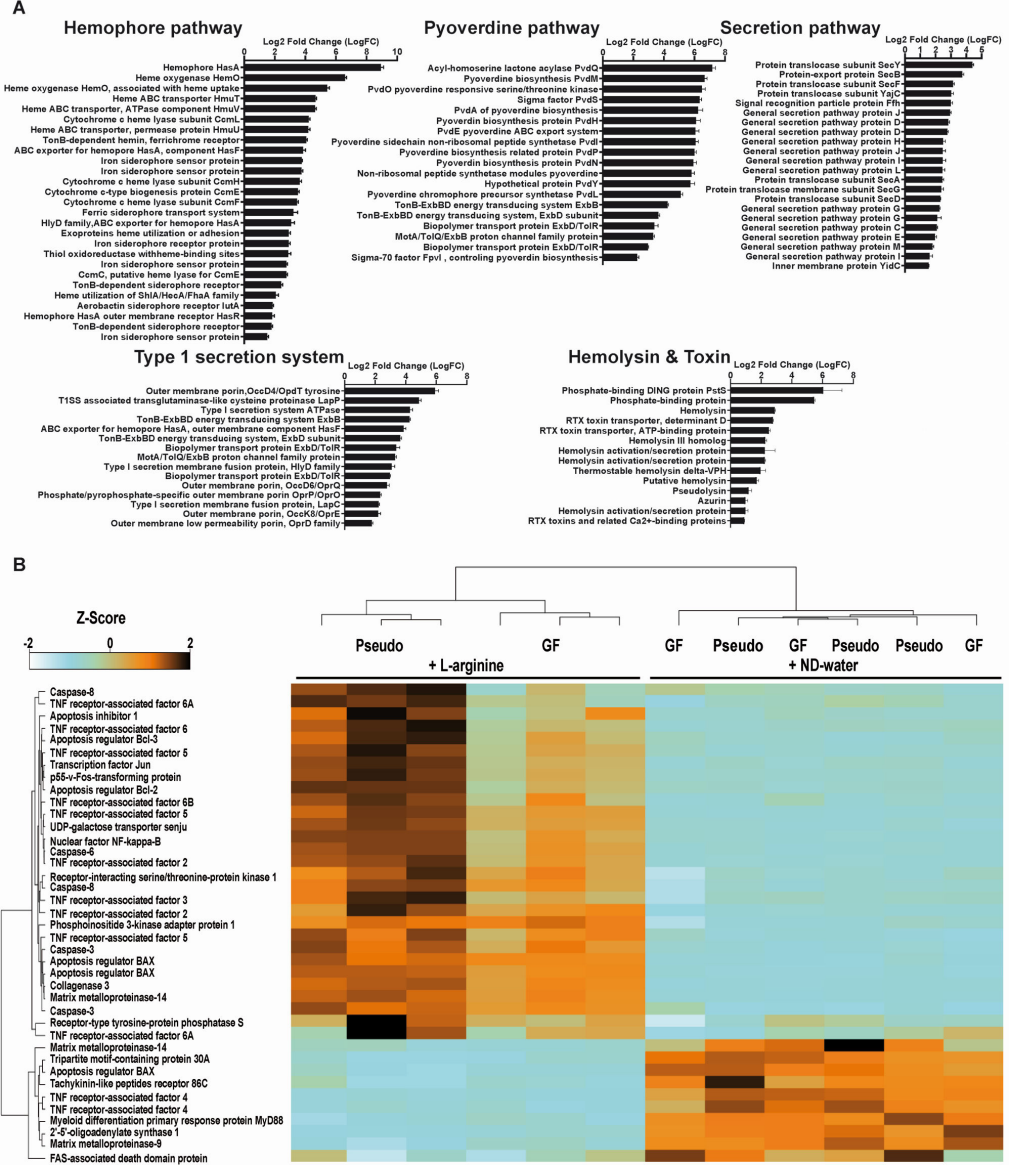
(**A**) Transcriptional changes of *Pseudomonas alcaligenes* T3. Bar chart illustrating transcriptional changes in *Pseudomonas alcaligenes* T3 when exposed to L-arginine, compared to *Pseudomonas* in nutrient-deficient water. (**B**) Heatmap of immune response genes: the heatmap specifically focuses on differentially expressed immune genes in *Hydra*, mono-colonized with *Pseudomonas* (Pseudo) and exposed to L-arginine, belonging to the interleukin and mitogen-activated protein kinase pathways (based on *Z*-score values). Germ-free (GF) polyps and nutrient-deficient water (ND-water) served as controls. Data are mean ± SE.

The supplementation of L-arginine resulted in a significant alteration of the transcriptional profile of *P. alcaligenes* T3 (Fig. 5A). Genes associated with iron-acquisition represented the majority of differentially regulated transcripts. Pyoverdine biosynthesis and transport systems were upregulated sixfold when *P. alcaligenes* T3 was exposed to L-arginine instead of nutrient-deficient water. In addition to other iron-scavenging proteins, the expression of hemophore HasA was increased ninefold. The expression of Type I and Type IV secretion systems was upregulated, accompanied by an increased production of virulence factors, including the DING protein, PstS, hemolysins, RTX toxins, and azurin (Fig. 5A).

#### Transcriptional changes during disease onset in *Hydra*

Disease onset in *Hydra* was already apparent due to initial tentacle shrinkage after 24 h. Compared to GF polyps in L-arginine, transcriptional analysis of *P. alcaligenes* T3 mono-colonized polyps exposed to L-arginine revealed a high proportion of differentially expressed apoptotic, necrotic, and immune response genes. Among those, several genes belonging to the interleukin and mitogen-activated protein kinase (MAPK) pathways were upregulated (Fig. 5B).

Another notable subset of genes with differential expression consisted of those encoding G proteins (Fig. S15). Among these, two DKF2 genes and two RAS1 genes (activators of MAPK) were upregulated. It is noteworthy that the elevation in gene expression was not limited to those with an immunostimulatory effect. Genes inhibiting the release of pro-inflammatory mediators, such as ADRB2, were observed to be elevated as well. Furthermore, the expression of small heat shock proteins such as CRYAB genes, which have been demonstrated to possess antiapoptotic activity, was increased (Fig. S16).

## DISCUSSION

This study examines two important aspects of host-microbe biology. The first is the influence of the host’s environmental context on the composition of its associated microbial communities. The second focuses on the environmental conditions that promote the transition from a symbiont to a lethal pathobiont.

### Both biotic and abiotic factors shape host-associated microbial communities

Our results contribute to the growing body of literature examining the interaction between host-associated and environmental microbiota in aquatic organisms (reviewed in reference 31). A previous study of *Hydra* polyps found that host-microbiota diversity was most strongly dependent on sampling location, indicating that the source environment plays an important role in the assembly process of the microbiota (32). Our observations with laboratory-grown *Hydra* indicate that exposure of polyps to different lake environments resulted in the colonization of new microbes and led to detectable shifts in community composition. In contrast to *Hydra* collected from the wild, laboratory cultures were acclimated to nutrient-deficient water for long periods of time in the absence of new colonizers. This environment likely creates a bottleneck, that favors microbes that are highly adapted to low-nutrient conditions and/or dependent on nutrients derived from *Hydra*.

Comparable patterns of reduced host microbial diversity have been observed in humans following antibiotic treatments (33) or prolonged exposure to sterile environments (34). Under such conditions, the presence of open niches would be expected to facilitate microbial colonization.

When *Hydra* polyps are exposed to different lake environments, the nutrient content creates a new selection pressure. This disrupts the established host-microbe interactions observed under nutrient-deficient conditions, allowing other microbes to colonize that are not specifically dependent on the nutrients provided by *Hydra*. In combination this leads to new colonization and detectable shifts in community composition. Exchange between host-associated bacteria challenges the view that host-specific microbial communities are stable and solely regulated and maintained by host immunity (13, 18, 19). Gacesa et al. (11) reached a similar conclusion and proposed that the environment, rather than the host, is the primary determinant of human microbiota composition. Comparable evidence has been uncovered in the microbiome of Atlantic salmon, which is influenced by the surrounding water (16). Additionally, observations in *Daphnia* indicate that environmental microbes alter host microbial community composition (35). Altogether, these findings support the proposition that the broader environment exerts a profound influence on an organism’s microbial community (36).

We subsequently postulated that environmental nutrients have the potential to disrupt host-microbe homeostasis, thereby facilitating uncontrolled microbial growth and the increase of microbial by-products that promote disease development (12). By removing microbes from the surrounding water and exposing *Hydra* polyps solely to the water chemistry of different lake environments, we were able to study the influence of lake water nutrients on the resident microbiota and compare it to a control group lacking additional nutrients. Our results suggest that the environment’s nutrient content can either inhibit or promote the growth of certain host-associated microbes. This phenomenon has been observed in corals (37) and other aquatic organisms (38). Moreover, changes in C:N and C:P ratios were shown to be associated with an increase or a decrease in specific microbial populations. These findings could be used to devise dietary compositions for a targeted manipulation of the microbiome.

Nutrient enrichment is a frequently studied environmental factor in coral reef ecosystems in relation to coral disease (39). Similar to these findings, we observed shifts *in Hydra*’s microbiome and the development of disease upon nutrient exposure. Our experiments demonstrate that external feeding of the microbial community undermines the competitive advantage of *Curvibacter* in the mucosal environment (40). Instead, external feeding favored microbes that were previously underrepresented in the natural mucus environment, thereby altering the host niche space and ultimately inducing dysbiosis. Furthermore, we observed a reduction in population growth as a consequence of dysbiosis, a phenomenon that has also been reported in corals, though the response may vary depending on the nutrient in question (41).

One of our key findings was that disease development and reduced population growth was linked to an increase in *Pseudomonas*. Our study identified the amino acid L-arginine as a critical substrate driving this increase in *Pseudomonas* abundance. These results suggest that L-arginine plays a key role in disturbing host-microbe homeostasis in *Hydra*.

### Harmful infection that originates within the body

L-arginine is a conditionally essential amino acid in host organisms playing a key role in many biological processes. These include cell division, wound healing, ammonia detoxification and immune function (42, 43). L-arginine also serves as a precursor for the synthesis of nitric oxide, an important antimicrobial component (44).

While previous studies have focused on L-arginine’s impact on host immune defense, information on the influence of metabolic changes in L-arginine metabolism on microbial pathogenesis is limited (45). To investigate this, *Hydra* polyps were mono-colonized with *Pseudomonas alcaligenes* T3 for the purpose of studying disease progression over time in the absence and presence of L-arginine. Interestingly, *Pseudomonas* alone was not sufficient for causing observable disease symptoms. Only in combination with L-arginine was disease development induced. Notably, L-arginine alone did not induce a disease phenotype in germ-free polyps, suggesting that while L-arginine itself is not harmful to *Hydra*, it may facilitate pathogenic interactions between *Pseudomonas* and the host.

L-arginine is a central metabolite for maintaining homeostatic host-microbe interactions, which is why the competition for L-arginine between host and microbiota needs to be carefully regulated. In mammals, sufficient levels of intra-luminal L-arginine can facilitate the restoration of the intestinal microbiota and the resolution of inflammatory or infectious processes. Conversely, deficiencies prolong bacterial persistence and perpetuate chronic inflammation (45). The importance of L-arginine metabolism in pathogenesis as a source of energy for bacteria during infection has been demonstrated in several organisms, including *Salmonella Typhimurium*, *Helicobacter pylori* and *Mycobacterium tuberculosis* (44).

At the same time, L-arginine can also function as a trigger for the expression of various pathogenicity genes (44), inducing the transition of *Pseudomonas* from a non-pathogenic to a pathogenic state. This transition was confirmed in our study by the upregulation of virulence-associated Type I and Type IV secretion systems and the production of virulence factors such as the DING protein, PstS, hemolysins, RTX toxins and azurin. Based on these transcriptional changes and the expression of iron-acquisition genes, we postulate that L-arginine caused a nutritional imbalance in *P. alcaligenes* T3 due to an excess of nitrogen and carbon, forcing it to sequester iron and phosphate from its environment as a means of compensation. The increased secretion of iron- and phosphate-binding metabolites, as well as various hemolysins and toxins known to disrupt eukaryotic cells, made *Pseudomonas* harmful to *Hydra*. As such, the host-bacterial equilibrium was shifted along the parasite-mutualist continuum (46) from a non-pathogenic to a pathogenic state. This is another example of the pathogenic potential (PP) concept, which states that all microbes have some degree of pathogenic potential (47). According to the PP concept, any microbe can cause disease if it is acquired by the host in sufficient quantities to surpass a certain threshold that affects homeostasis and causes damage.

The observed upregulation of interleukin and MAPK pathways in *Hydra* likely contributed to the tissue regeneration observed in *Pseudomonas-*induced degradation processes (48). DKF-2 activation in polyps is essential for the induction of immune effector mRNAs encoding antimicrobial peptides and its increased expression has been shown to protect *C. elegans* after ingestion of *P. aeruginosa* strain 14 (PA14) (49). Genes that stop the release of pro-inflammatory mediators, such as ADRB2, were also found to be more active. These genes have been shown to play an important role in the pathogenesis of asthma (30). Furthermore, the expression of CRYAB, a small heat shock protein with antiapoptotic activity, was increased. These compensatory responses were likely implemented by *Hydra* to counteract *Pseudomonas*-induced apoptosis and were enhanced by the upregulation of three carbonic anhydrases (CAHs). CAHs are involved in the acidification of the human gut environment and are effectors of the innate immune response that regulates bacterial infections (31, 32). These innate immune responses and defense mechanisms have an energy cost. Consequently, polyp shrinkage may indicate a reallocation of resources from *Hydra* body mass to host defense, suggesting that this is a consequence of disease development.

In conclusion, this study highlights the link between the host microbiome and the environment in which organisms live. In order to gain a comprehensive understanding of organismal health, it is essential to monitor microbial trafficking at the ecosystem level between the environment and the host. In addition, elevated concentrations of a single amino acid were observed to transform a symbiont into a lethal pathobiont, providing empirical support for the pathogenic potential concept, which postulates that there is no clear dividing line between pathogenic and non-pathogenic microbes.

## MATERIALS AND METHODS

### Long-term laboratory *Hydra* culture conditions

*Hydra vulgaris* (AEP) (50) was cultured under constant laboratory conditions in nutrient-deficient water (ND-water) (0.28 mM CaCl_2_, 0.33 mM MgSO_4_, 0.5 mM NaHCO_3_, and 0.08 mM KCO_3_) at 18°C water temperature and a 12 h light-dark cycle, according to standard procedure (51).

#### Exposing laboratory-grown *Hydra* to natural lake environments

##### Field experiment

Laboratory-grown *Hydra vulgaris* (AEP) polyps with their associated bacterial community were exposed to different lake environments for a period of up to 7 days. *Hydra* polyps were relocated to three mesotrophic lakes (Schluensee, Selenter See, and Plußsee) and three eutrophic lakes (Lanker See, Postsee, and Tresdorfer See) (Fig. S1). To allow for polyp retrieval, 15 polyps per replicate were transferred into 50 mL falcon tubes (*n* = 5). The openings were sealed with gauze (150 µm) to allow exchange with the surrounding environment and influx of bacteria while preventing immigration of zooplankton and polyp loss. Tubes containing polyps were tied together and deployed at 30 cm water depth. After 1, 4, and 7 days, one polyp per treatment and replicate (*n* = 5) was collected. Polyps were transferred separately to 1.5 mL tubes, washed twice in 500 µL sterile ND-water, and stored at −20°C until DNA extraction.

##### Laboratory experiment

In parallel to the field experiment, a similar experiment was carried out under controlled laboratory conditions at 18°C water temperature and a 12 h light-dark cycle. Lake water was collected from different lake environments (see above) and filtered through 1.5 µm to remove larger organisms and particles. *Hydra* polyps were transferred separately into six-well plates. Five polyps per replicate (*n* = 6) were exposed to the six different lake water environments. On days 1, 4, and 7, one polyp per replicate was collected in a 1.5 mL tube, washed twice in 500 µL sterile ND-water, and stored at −20°C until DNA extraction.

##### Exposure of laboratory-grown *Hydra* to lake nutrients

Lake water was filtered at 0.02 µm in order to remove all bacteria and phages to test the effect of the chemical environment on *Hydra*’s microbial community composition. The nutrient composition of the lake water, including DOC, total nitrogen and total phosphate, was analyzed by the Landeslabor Schleswig-Holstein and Landesamt für Landwirtschaft, Umwelt und ländliche Räume (LLUR) (Table S1). Water was changed daily. After 7 days of exposure, polyps were collected and prepared for DNA extraction (see below).

### Nutrient manipulation of the environment

#### Exposure to complex nutrients

The effect of elevated nutrients on *Hydra* host-microbe homeostasis was tested by adding either a protein source (P+) or a complex nutrient source (C++) to ND-water. P+ was prepared by adding 0.05 mg/mL peptone, while C++ was made using bacterial culture medium R2A (ROTH) at a final concentration of 0.3 mg/mL. To control for the effect of nutrients on the eukaryotic host in the absence of associated bacteria, GF and wt polyps were exposed separately to ND-water conditions. Polyps exposed to ND-water served as controls. GF *Hydra* polyps were generated by exposing polyps to an antibiotic cocktail containing 50 µg/mL of ampicillin, rifampicin, streptomycin, spectinomycin, and neomycin for a period of 2 weeks (29). Antibiotic solutions were exchanged every second day. To remove remaining antibiotics, animals were transferred to antibiotic-free, sterile ND-water for 3 days after antibiotic treatment. Sterility was confirmed by negative bacterial 16S PCR (17). The effect of nutrients on the host-associated microbial community was analyzed every 24 h (*n* = 5). Polyps were washed three times and finally homogenized in 500 µL sterile ND-water. One hundred microliters was plated onto R2A agar (ROTH) plates to establish a bacterial culture collection that served as a reservoir of potential bacterial pathogens. Two hundred microliters was used for DNA extraction using the DNA Blood and Tissue Kit (Qiagen). Microbial community composition was determined by rRNA gene amplicon sequencing (see below).

#### Evaluation of disease symptoms

Disease development in *Hydra* is typically measured by scoring morphological changes (28, 29). As disease development starts with shrinking tentacles, we measured tentacle length every 24 h for a period of 4 days (*n* = 12) to access early signs of morphological changes. Polyps were photographed using a binocular microscope, and length estimates were made using ImageJ 1.50i (52). Severe disease states were assessed through symptom grading. The severity was determined using a numerical scale, starting with a score of 0 for healthy polyps. Scores progressed at a reduction in tentacle length and eventual lysis of polyp tissue (scores 3–1). Total tissue degradation, accompanied by the loss of polyp body shape, was scored as 9.

#### Population growth experiment

To test the effect of elevated nutrient concentrations corresponding to eutrophic natural lake environments, *Hydra* polyps were individually transferred to six-well plates (*n* = 6) containing 0.03 mg/mL R2A. Population growth was estimated by counting bud production over a period of 47 days. At the end of the experiment, polyps were removed, washed in sterile ND-water and stored at −20°C until DNA extraction.

#### Biolog experiment

In total, 1,152 *Hydra vulgaris* (AEP) polyps were washed and kept in sterile ND-water for 3 days without feeding prior to exposure to Biolog plates. The compounds in the Biolog plates (PM1 MicroPlate Carbon Sources, PM2A MicroPlate Carbon Sources, PM3B MicroPlate Nitrogen Sources, and PM4A MicroPlate Phosphorus and Sulphur Sources with replication [*n* = 3]) were dissolved in sterile ND-water and diluted 20-fold. Polyps were randomly distributed into Biolog plates, placing one polyp per well. After 48 h of exposure, polyps were removed, washed twice in sterile ND-water, and then stored at −20°C until DNA extraction.

#### Recolonization experiment

Germ-free *Hydra* polyps were mono-colonized with *Pseudomonas alcaligenes* T3. Five thousand CFUs/mL was added into the surrounding water of GF polyps and incubated for 1 day. Non-attached bacteria in the surrounding medium were removed by washing the polyps in sterile ND-water. Mono-colonized polyps and GF polyps (*n* = 5) were exposed to either L-arginine or ND-water. Disease development was evaluated by scoring morphological changes (28, 29).

#### Microbial community analysis

Two hundred microliters of homogenized polyps (see above) was used for DNA extraction (DNA Blood and Tissue Kit, Qiagen). Microbial community composition was determined by amplicon sequencing of the variable region V1–V2 of the 16S rRNA gene. We used the following primers: forward primer 27F: (5′-AATGATACGGCGACCACCGAGATCTACAC XXXXXXXX TATGGTAATTGT AGAGTTTGATCCTGGCTCAG-3′) and reverse primer 338R: (5′-CAAGCAGAAGACGGCATACGAGAT XXXXXXXX AGTCAGTCAGCC TGCTGCCTCCCGTAGGAGT-3′). Primers contained the Illumina adapter p5 (forward) and p7 (reverse) and unique MIDs (designated as XXXXXXXX) to label each PCR product. PCR reactions were performed in duplicate using Phusion Hot Start DNA Polymerase (Finnzymes, Espoo, Finland). PCR cycling conditions were 98°C for 30 s, 30 × (98°C, 9 s; 55°C, 30 s, and 72°C, 90 s), 72°C, 10 min. PCR products were combined and purified by using the MinELute Gel Extraction Kit (Qiagen) after agarose gel electrophoresis. Sequencing was performed on the Illumina MiSeq platform at the sequencing facility of the Kiel Institute for Clinical Molecular Biology (IKMB). Sequencing data were analyzed using the MOTHUR packages (53) according to the MiSeq SOP (54). In summary, MiSeq paired-end reads were assembled and quality-controlled resulting in 12,028 sequences per sample. Sequences were grouped into operational taxonomic units (OTUs) using a 97% similarity threshold. Sequences were aligned to the SILVA 128 database and taxonomically classified by the RDP classifier. Multidimensional scaling analysis of OTU abundance data based on Bray-Curtis similarity was performed by the Primer software v.7.0.13 (Primer-E) (55). Similarities between different treatment groups were analyzed by analysis of similarity (ANOSIM). ANOSIM pairwise test *R* values close to 1 indicate that group similarity is higher within compared to in between different groups. Raw data are deposited in the Sequence Read Archive (SRA) and are available under project ID PRJNA997121, SAMN36665612–SAMN36665853 (lake environment), SAMN36666071–SAMN36666115 (complex nutrients), and SAMN37278454–SAMN37279040 and SAMN37280028–SAMN37280584 (Biolog).

#### *Pseudomonas alcaligenes* T3 genome sequencing and annotation

The Nextera XT kit (Illumina) was used for library preparation. Bacterial DNA was 2 × 150 bp paired-end sequenced on a NovaSeq platform (Illumina) at the IKMB in Kiel. Raw Illumina reads were adapter-trimmed, quality-trimmed, and filtered using BBTools v.38.96 (56) and fastp v.0.23.2 (57). A long-read library was made using the Rapid Sequencing Kit (SQK-RAD004) and sequenced on the MinION (Oxford Nanopore Technologies, Oxford, UK) with a Flongle flow cell (FLO-FLG001). The super-accurate model of Guppy (v.6.2.1+6588110, dna_r9.4.1_450bps_sup; Oxford Nanopore Technologies PLC) was used for base-calling, and reads were adapter-trimmed with Porechop v.0.2.4 (58). A hybrid genome assembly, using short and long-reads, was generated with Unicycler v.0.5.0 (59), and its completeness was evaluated with CheckM v.1.2.2 (60) and BUSCO v.5.4.3 (61). The genome sequence of *Pseudomonas alcaligenes* T3 is available under project ID PRJNA997121, SAMN37208214.

#### Phylogeny

Taxonomic classifications were performed with GTDB-Tk v.2.2.6 (62) using the classify and *de novo* workflow with *Pseudomonas* reference genomes from the Genome Database Taxonomy (GTDB, r207) (62). GTDB-Tk identified T3 as *P. alcaligenes* (GCF_000474255.1) based on an average nucleotide identity of 98.64%. A *de novo* pipeline was used to calculate a phylogenetic tree and to confirm genome placement and identification. For this, genomes from *Pseudomonas* groups F and O (as outgroup) were used in the alignment.

#### *Hydra* RNA extraction

GF and mono-colonized polyps with *Pseudomonas alcaligenes* T3 were exposed to either ND-water or L-arginine (*n* = 3). In total, 15 polyps per replicate (*n* = 3) were incubated under these conditions for 24 h. Polyps were collected and dissolved in 750 µL TRIzol at room temperature and stored at −80°C. Upon thawing, 250 µL chloroform was added; samples were mixed and incubated at room temperature for 5 min. Samples were centrifuged at 12,000 × *g* for 15 min at 4°C. The upper phase was transferred into a new tube, mixed with 1× volume of cold ethanol, and transferred into spin cartridges. Subsequent purification steps were conducted according to the PureLink RNA Mini Kit (Thermo Fisher Scientific), except that all washing steps were performed twice. RNA was eluted into 35 µL RNase-free water and stored at −80°C prior to sequencing.

#### *Hydra* RNA-seq and data analysis

Strand-specific cDNA libraries were prepared using TruSeq adapters and sequenced (paired-end) via NovaSeq 6000 S4 PE150 XP RNA. Sequences were analyzed according to Batut et al. (63). In brief, we used Cutadapt (64), Trimmomatic (65), FastQC (66), and MultiQC (67) for quality control. We used RNA Star (68) to map reads to the *Hydra vulgaris* (AEP) genome provided by Cazet et al. (69). Featurecounts (70) was used to count reads. Finally, we conducted differential gene expression analysis via Deseq2 (71). The raw data are deposited at the SRA and are available under project ID PRJNA997121, SAMN37352379–SAMN37352390.

#### Bacterial RNA-seq

*Pseudomonas alcaligenes* T3 was exposed to either ND-water or L-arginine with replication (*n* = 4). After 12 h of exposure, bacterial cells were harvested by centrifugation, and bacterial RNA was extracted according to the method described above. Illumina Stranded Total RNA Prep was used for ribosomal removal and cDNA library preparation. Sequencing was conducted on a NovaSeq 6000 platform (Illumina). Sequence reads were trimmed and adapters were removed using Trimmomatic-0.36 (65). Trimmed and quality-controlled reads were mapped separately against the genome of *P. alcaligenes* T3 using Bowtie2 (72) and SAM tools (73). Coverage was calculated and normalized to the housekeeping gene *rpoS* (74). Raw data are deposited in the SRA and available under project ID PRJNA997121, SAMN37352367–SAMN37352374.

## Data Availability

All data supporting the findings of this study are available within the paper and its supplemental information. Sequence data are deposited in the National Center for Biotechnology Information and available under the project ID PRJNA997121.

## SUPPLEMENTAL MATERIAL

Supplemental material mbio.03843-24-s0001.pdf Fig. S1-S16; Table S1.

ASM does not own the copyrights to Supplemental Material that may be linked to, or accessed through, an article. The authors have granted ASM a non-exclusive, world-wide license to publish the Supplemental Material files. Please contact the corresponding author directly for reuse.
