# Supplementary material for: Nutrition-induced changes in the microbiota can cause dysbiosis and disease development
Source: mBio. 2025 Feb 25;16(4):e03843-24. doi: 10.1128/mbio.03843-24 (PMC11980362; doi:10.1128/mbio.03843-24)
Supplement: Supplemental material — Fig. S1-S16; Table S1. [file mbio.03843-24-s0001.pdf]

Supplemental Material

Nutrition induced changes in the microbiota can cause dysbiosis and disease development

Tim Lachnit[1]*†, Laura Ulrich[1], Fiete M. Willmer[1], Tim Hasenbein[2], Leon X. Steiner[3], Maria Wolters[4], Eva M. Herbst[1], Peter Deines[1]*

Corresponding author: tlachnit@zoologie.uni-kiel.de; t.lachnit@web.de; pdeines@zoologie.uni-kiel.de

The PDF file includes:

Figs. S1 to S16
Tables S1

Supplementary Figures

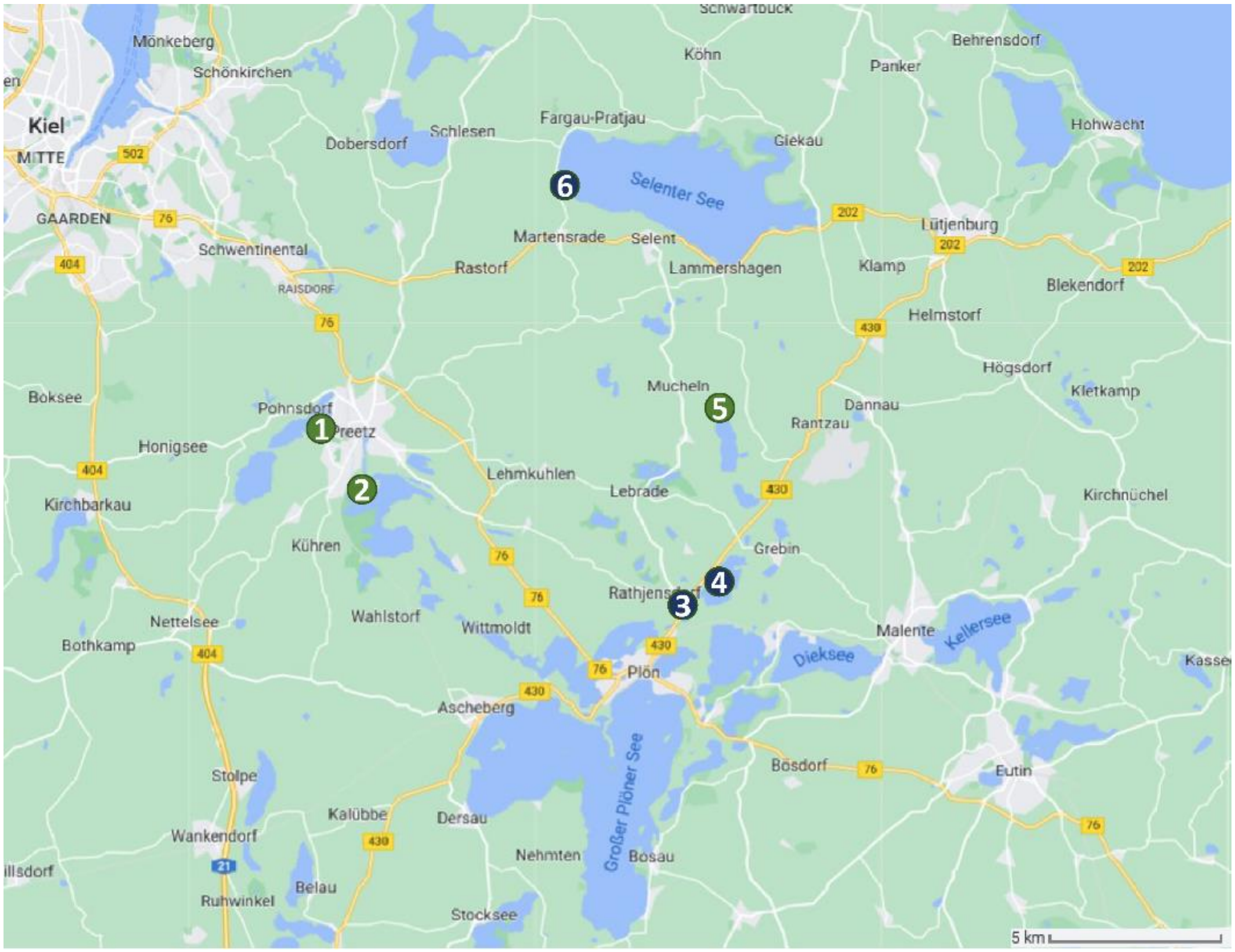

Fig. S1. Experimental sites. (1) Postsee=**PO** (eutroph), 24211 Pohnsdorf 54°14'05.5"N 10°15'46.3"E; 54.234867, 10.262865; (2) Lanker See=**LA** (eutroph), 24211 54°13'13.3"N 10°17'53.1"E; 54.220360, 10.298077; (3) Plußsee=**PL** (mesotroph), 24306 Rathjensdorf 54°11'02.4"N 10°26'32.9"E; 54.184006, 10.442480; (4) Schluensee=**SC** (mesotroph), 54°11'08.2"N 10°27'16.1"E; 54.185604, 10.454465; (5) Tresdorfer See=**TR** (eutroph), 54°13'51.5"N 10°27'55.7"E; 54.230980, 10.465476; (6) Selenter See=**SE** (mesotroph), 54°17'54.0"N 10°25'08.1"E; 54.298335, 10.418915.

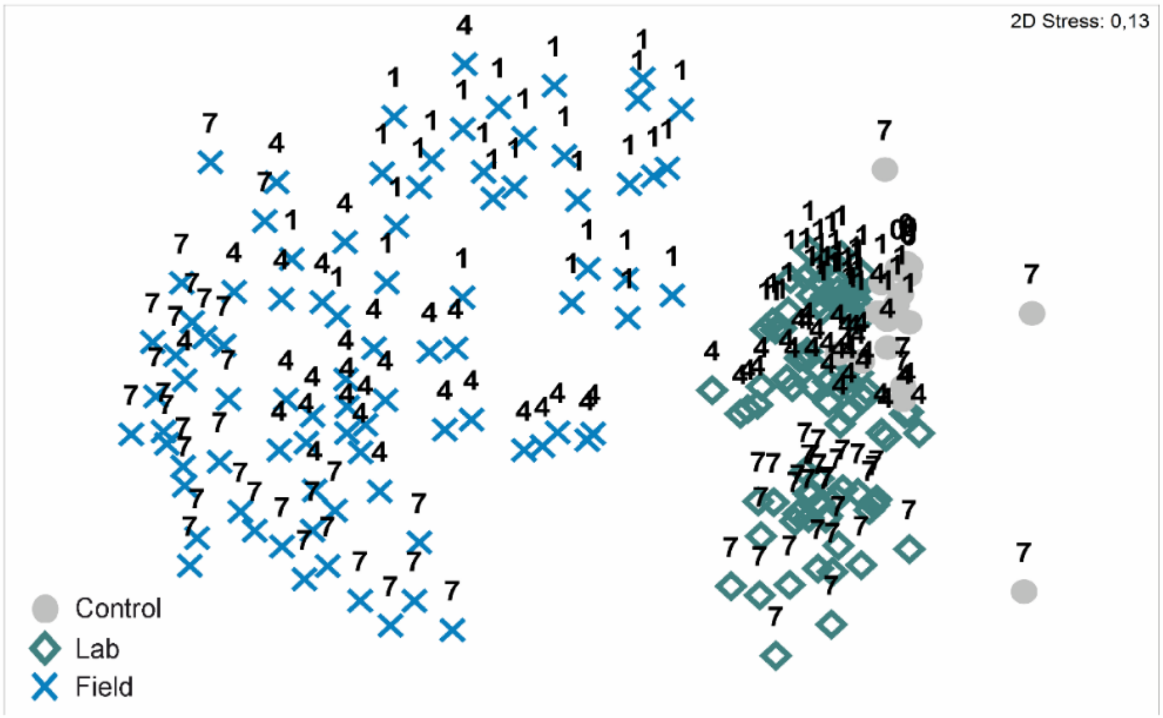

Fig. S2. nMDS analysis of *Hydra*-associated microbial community composition exposed to different lake water environments (laboratory and field) based on 16S rRNA gene amplicon sequencing. Nutrient-deficient water (ND-water) was used as control in the laboratory. Microbial community composition of control polyps was significantly differed from polyps exposed to different lake water environments (Permanova P<0.001).

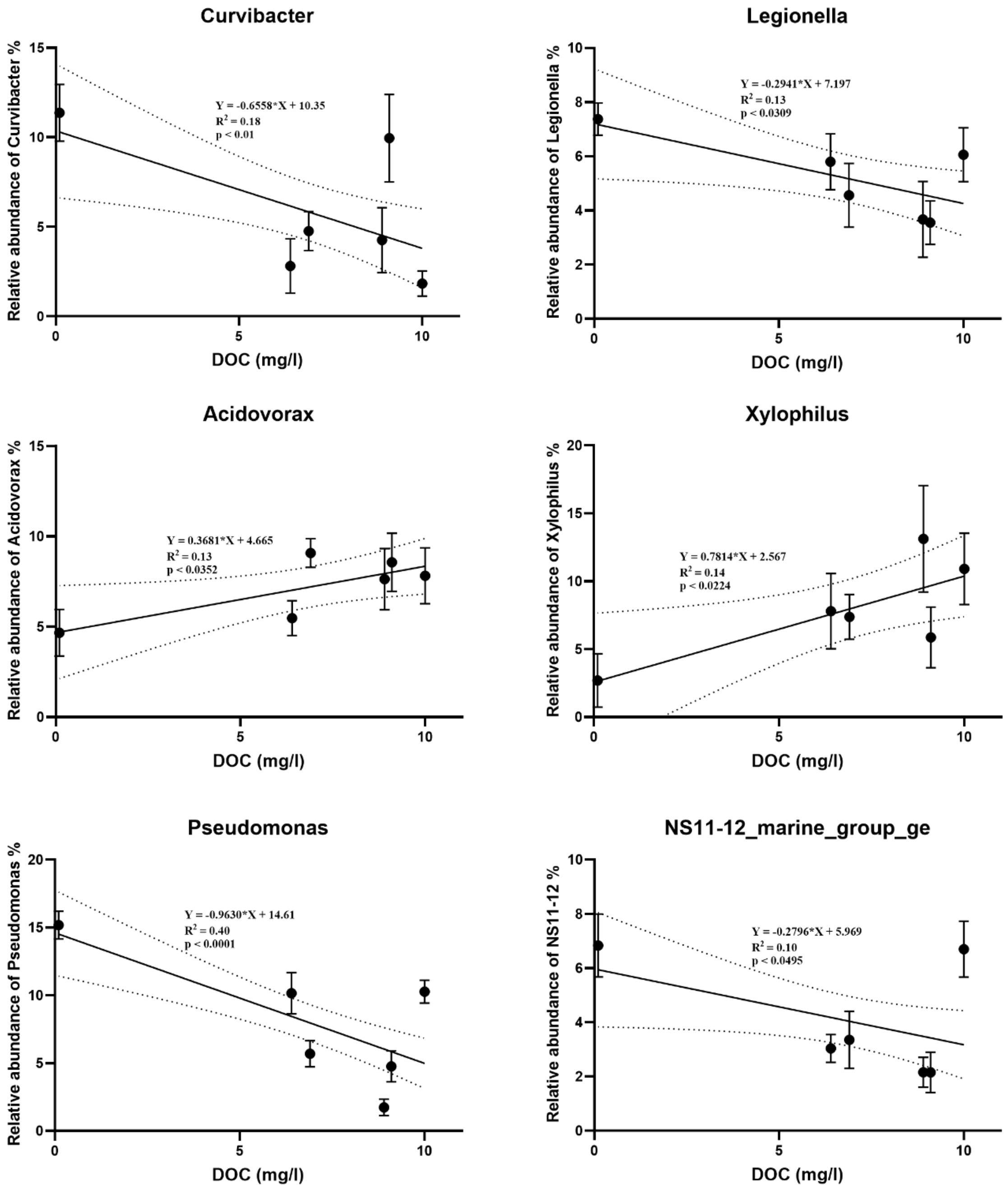

Fig. S3. Simple linear regression between the relative abundance of bacteria and dissolved organic carbon (DOC).

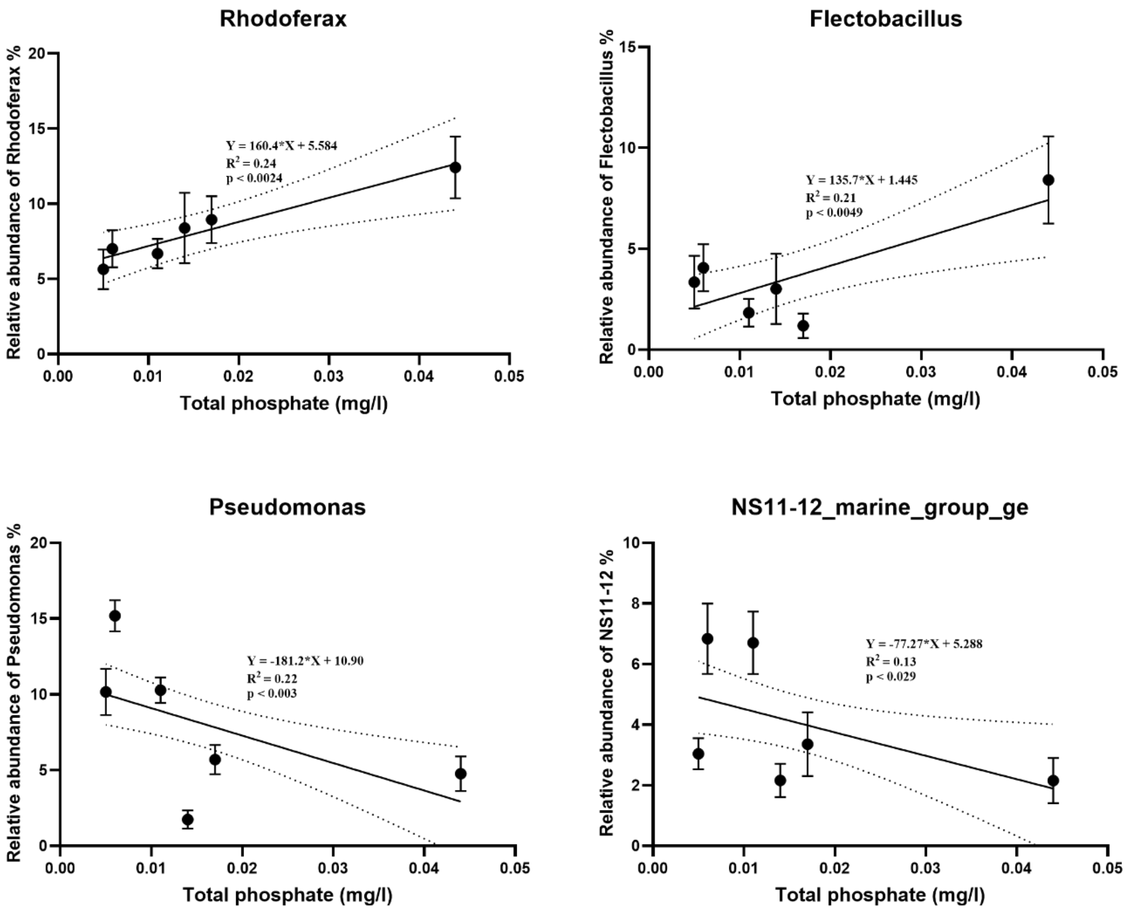

Fig. S4. Simple linear regression between the relative abundance of bacteria and total phosphate.

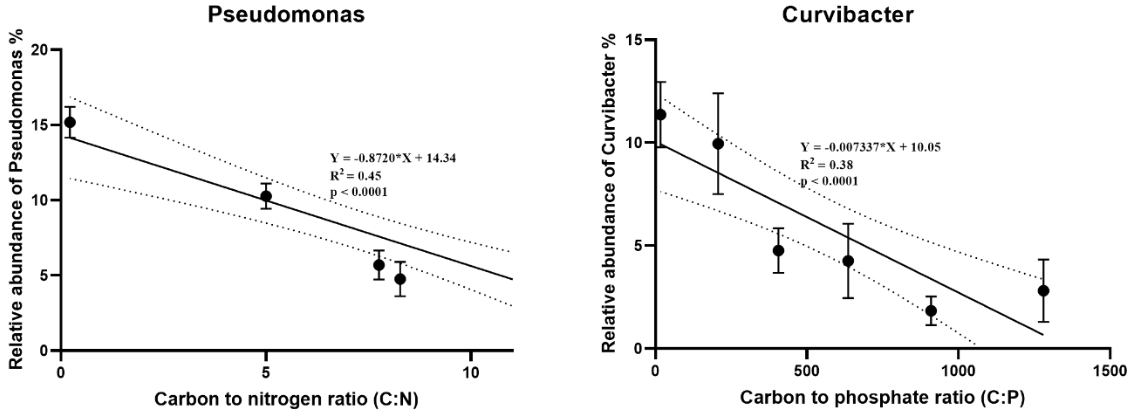

Fig. S5. Simple linear regression between the relative abundance of bacteria and nutrient ratios (C/N and C/P).

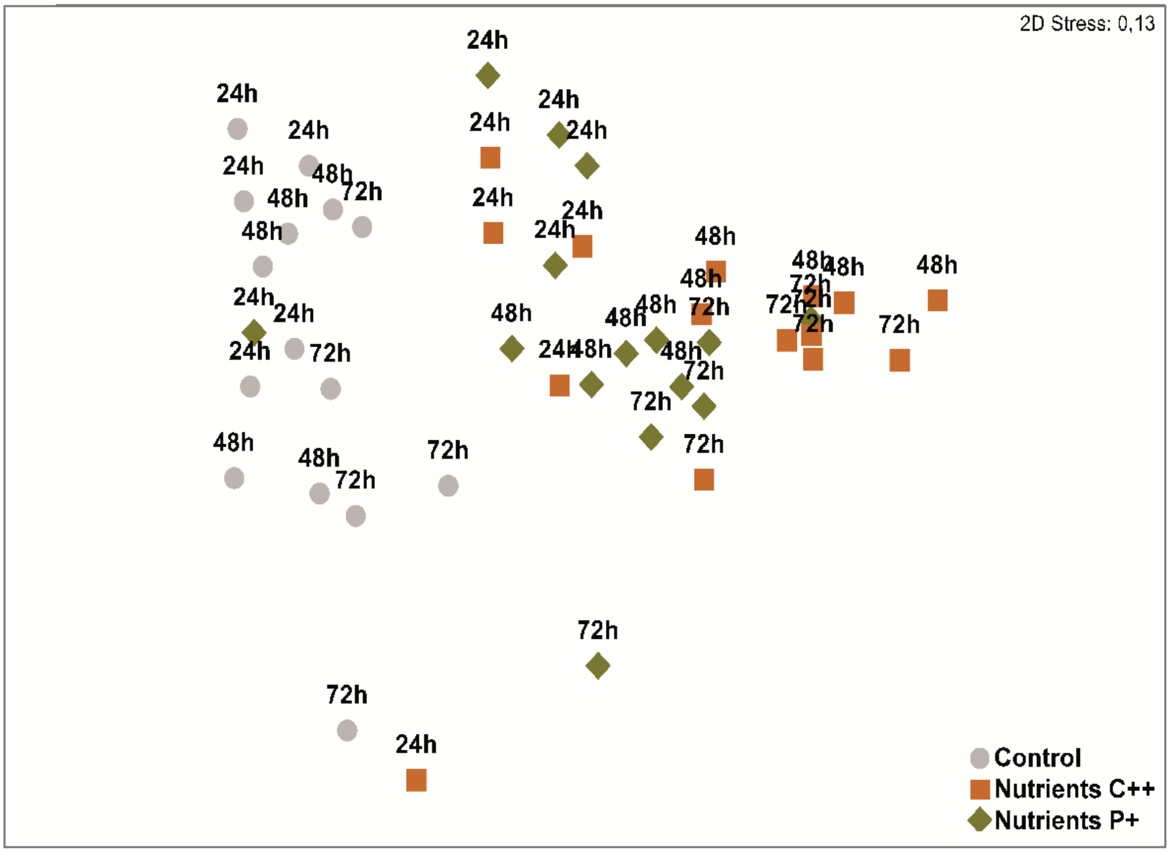

Fig. S6. External feeding of mucus-associated bacteria on *Hydra* altered the microbial community composition. Nonmetric multidimensional scaling (nMDS) analysis of 16S rRNA gene amplicon sequencing data revealed two distinct clusters. Polyps exposed to nutrient-enriched environments clustered separately from control polyps in nutrient-deficient water (grey circles). The microbial community composition of control polyps was significantly differed from polyps exposed to nutrient C++ (ANOSIM Pairwise-Test: R Statistic=0.776, P=0.001) and nutrient P+ (ANOSIM Pairwise-Test: R Statistic=0.832, P=0.001).

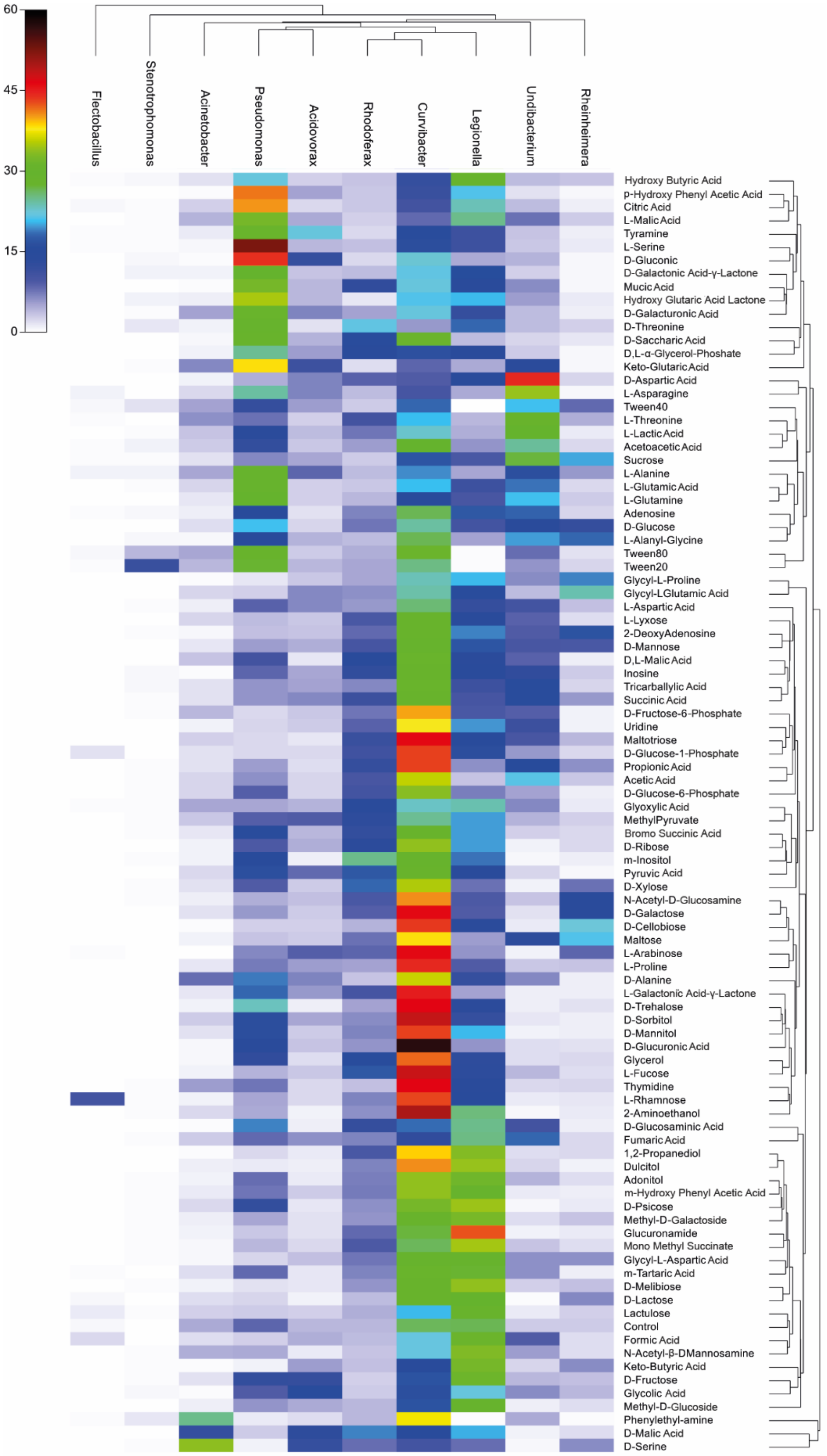

Fig. S7. Bacterial community shifts of *Hydra*-associated bacteria following a 48h exposure to different compounds of the PM1 MicroPlate™ (Carbon Sources). The color-scale heatmap illustrates the relative abundance of the most prevalent OTUs in percent.

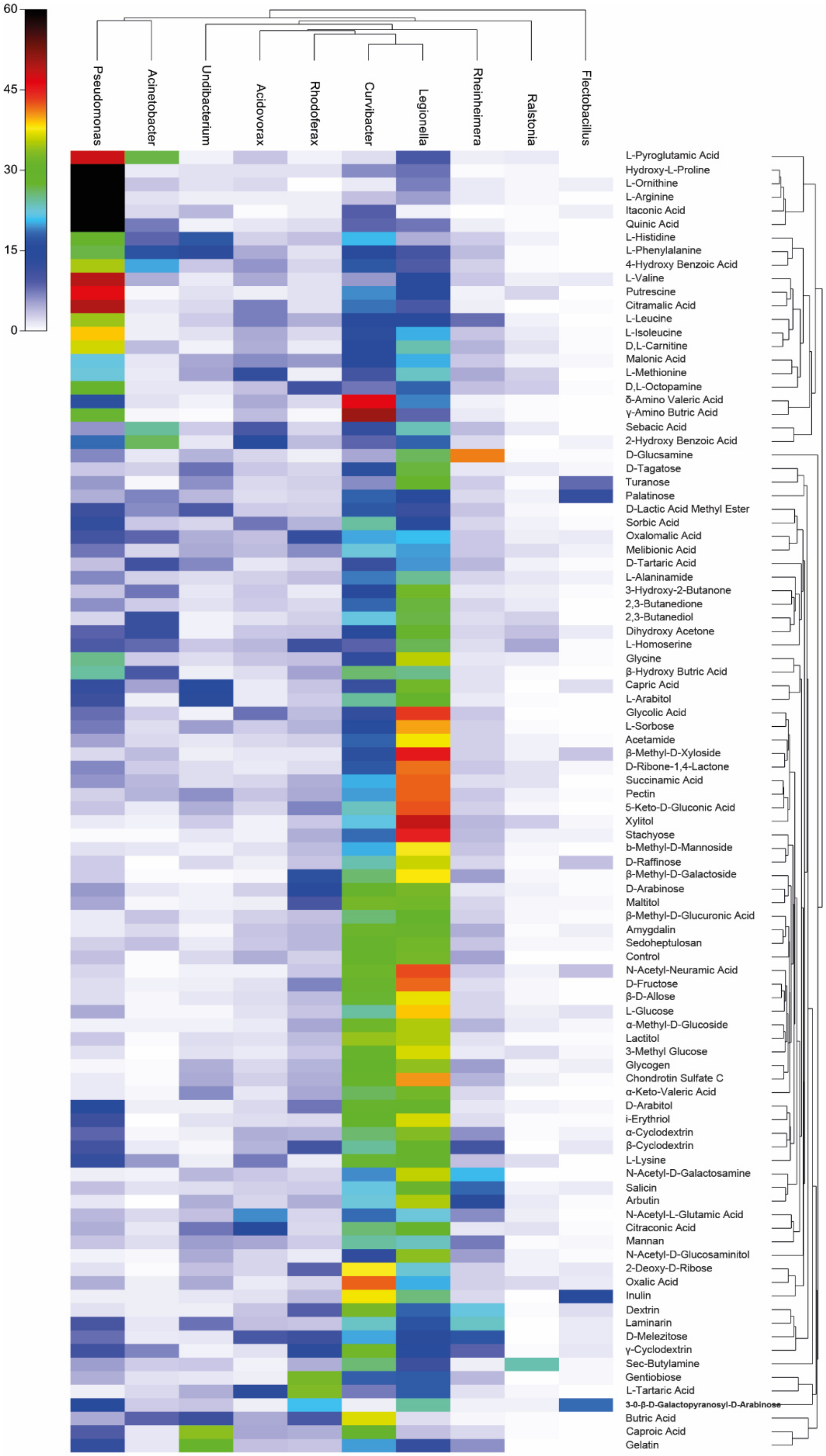

Fig. S8. Bacterial community shifts of *Hydra*-associated bacteria following a 48h exposure to different compounds of the PM2A MicroPlate™ (Carbon Sources). The color-scale heatmap illustrates the relative abundance of the most prevalent OTUs in percent.

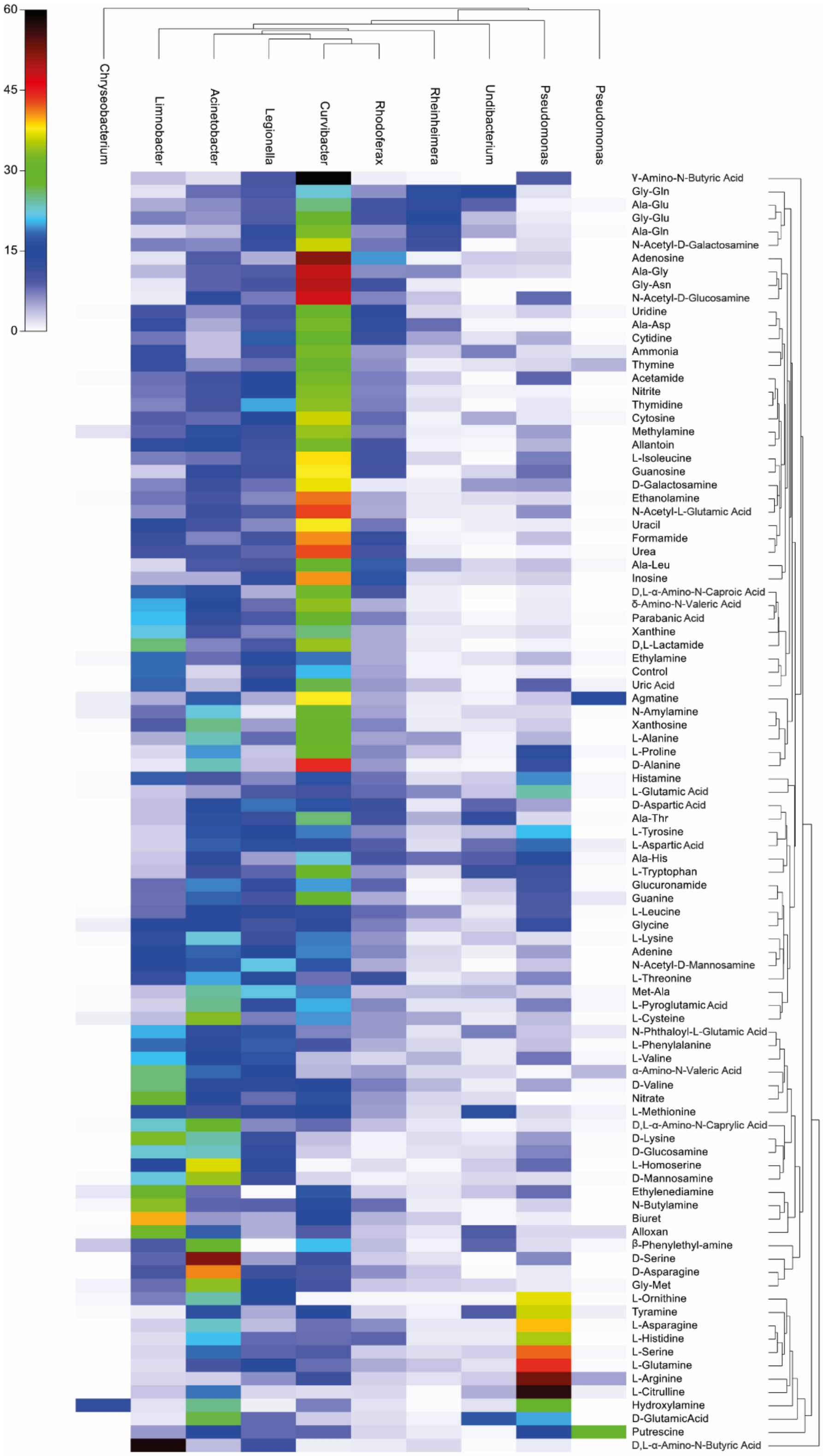

Fig. S9. Bacterial community shifts of *Hydra*-associated bacteria following a 48h exposure to different compounds of the PM3 MicroPlate™ (Nitrogen Sources). The color-scale heatmap illustrates the relative abundance of the most prevalent OTUs in percent.

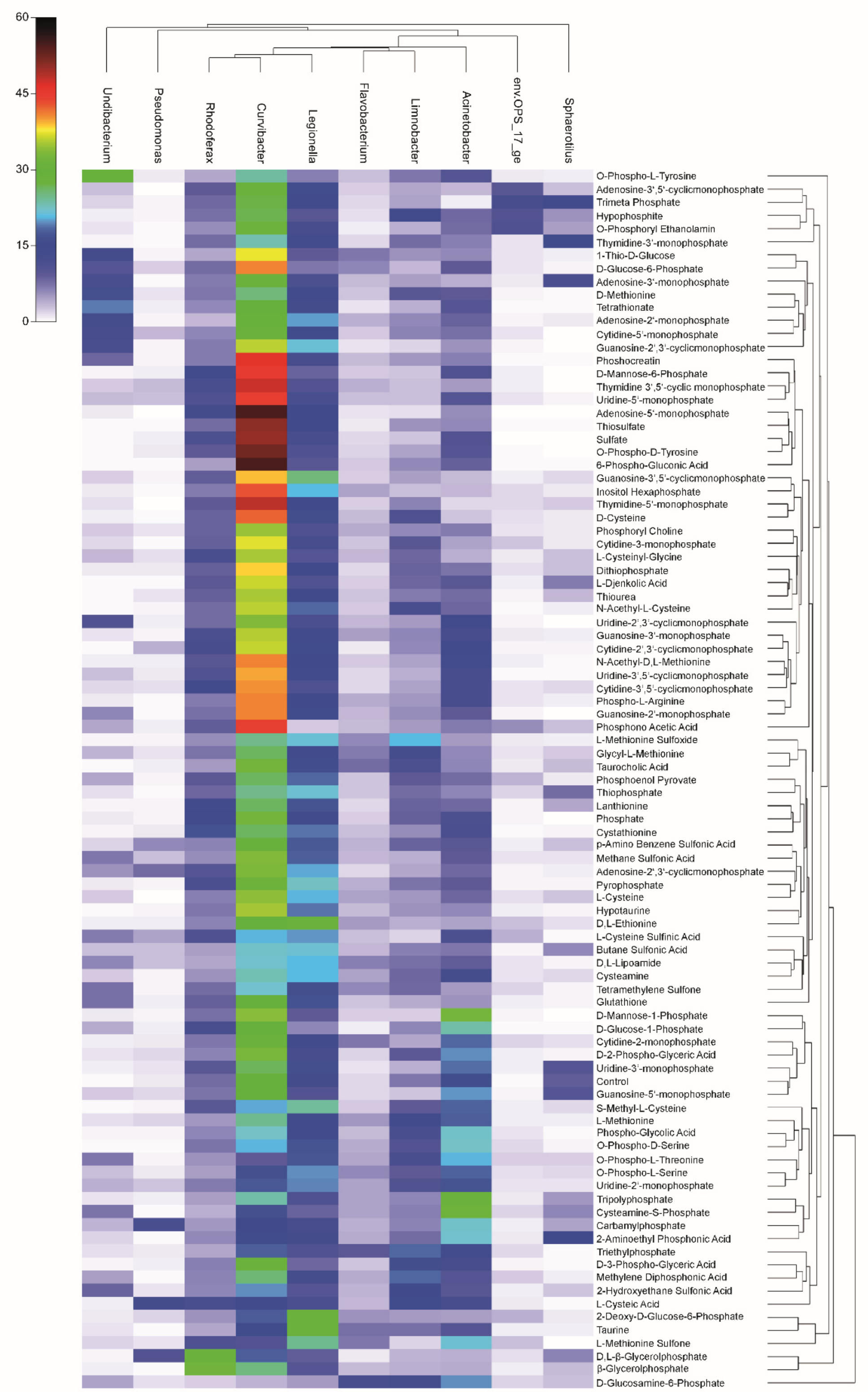

Fig. S10. Bacterial community shifts of *Hydra*-associated bacteria following a 48h exposure to different compounds of the PM4A MicroPlate™ (Phosphorus and Sulphur Sources). The color-scale heatmap illustrates the relative abundance of the most prevalent OTUs in percent.

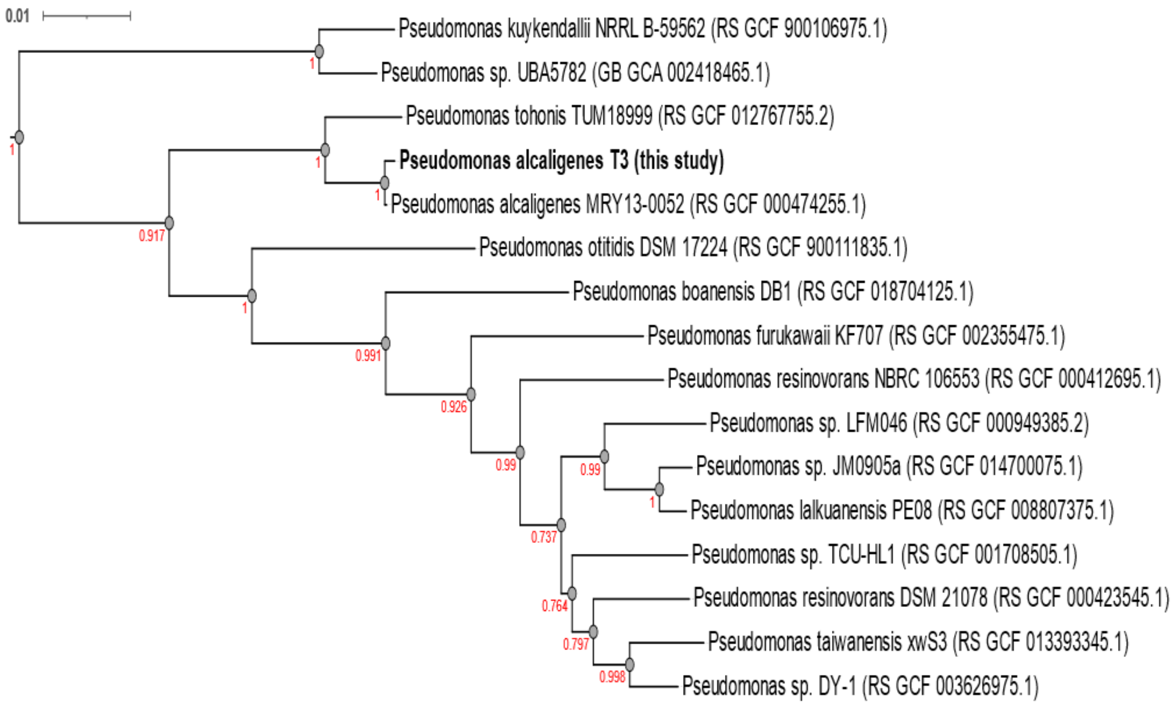

Fig. S11. Phylogenetic tree: Genome phylogeny of *Pseudomonas alcaligenes* T3 with reference genomes of *Pseudomonas* group F (and group O used as outgroups) in the Genome Taxonomy Database (GTDB). A maximum-likelihood phylogenetic tree is constructed based on 120 bacterial single-copy marker proteins. The bar indicates 0.01 substitutions per nucleotide position.

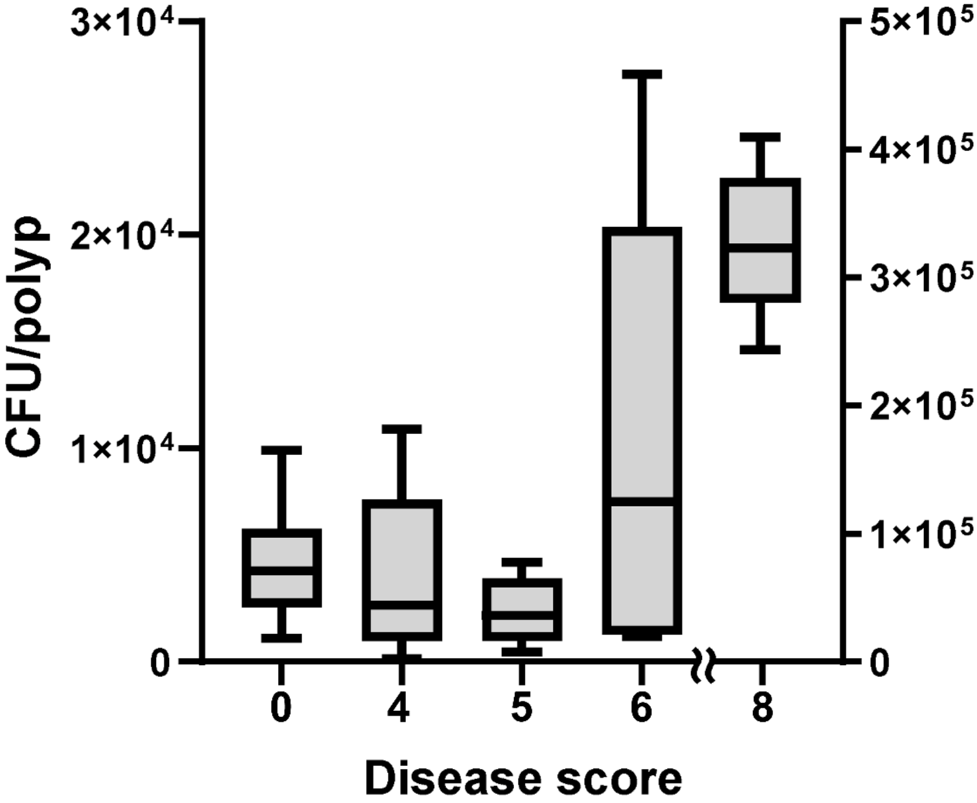

Fig. S12. *Pseudomonas* quantification per polyp (n=5) at different disease states was determined by counting colony forming units (CFUs). Mono-colonized, healthy polyps (score 0) in nutrient-deficient water (control) were compared to mono-colonized polyps exposed to L-arginine at disease scores 4 to 8 (n=5). The line in the box represents the mean. The whiskers max. and min. values and the box extents from 25-75 percentiles.

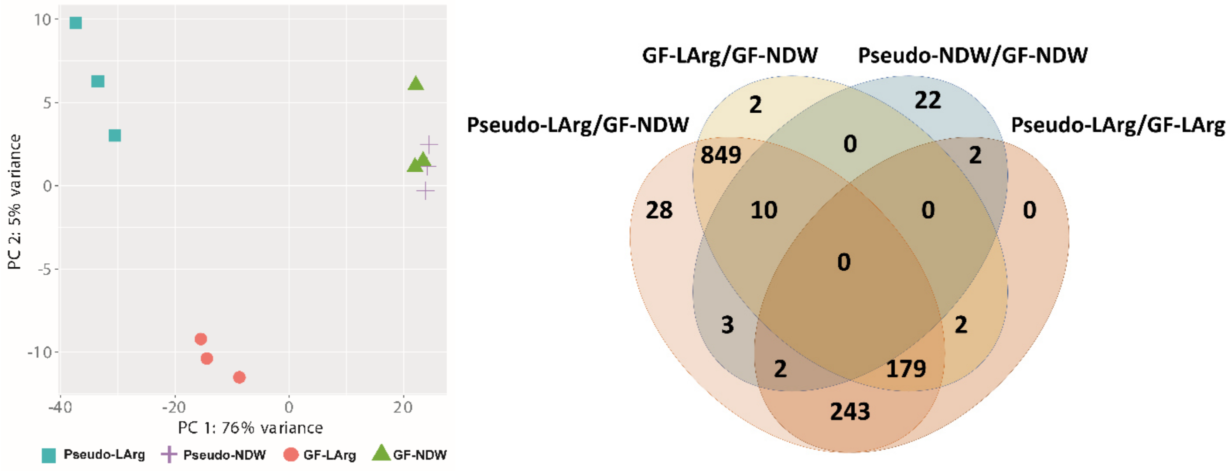

Fig. S13. Transcriptional analysis of diseased *Hydra* 24 h post exposure. (Left) Principle component analysis (PCA) plot illustrating transcriptional differences between mono-colonized (*Pseudomonas alcaligenes* T3 (Pseudo)) and germ-free (GF) *Hydra* polyps exposed to L-arginine (LArg) or nutrient-deficient water (NDW). (Right) Venn-diagram illustrating differential regulation of annotated genes in different treatments.

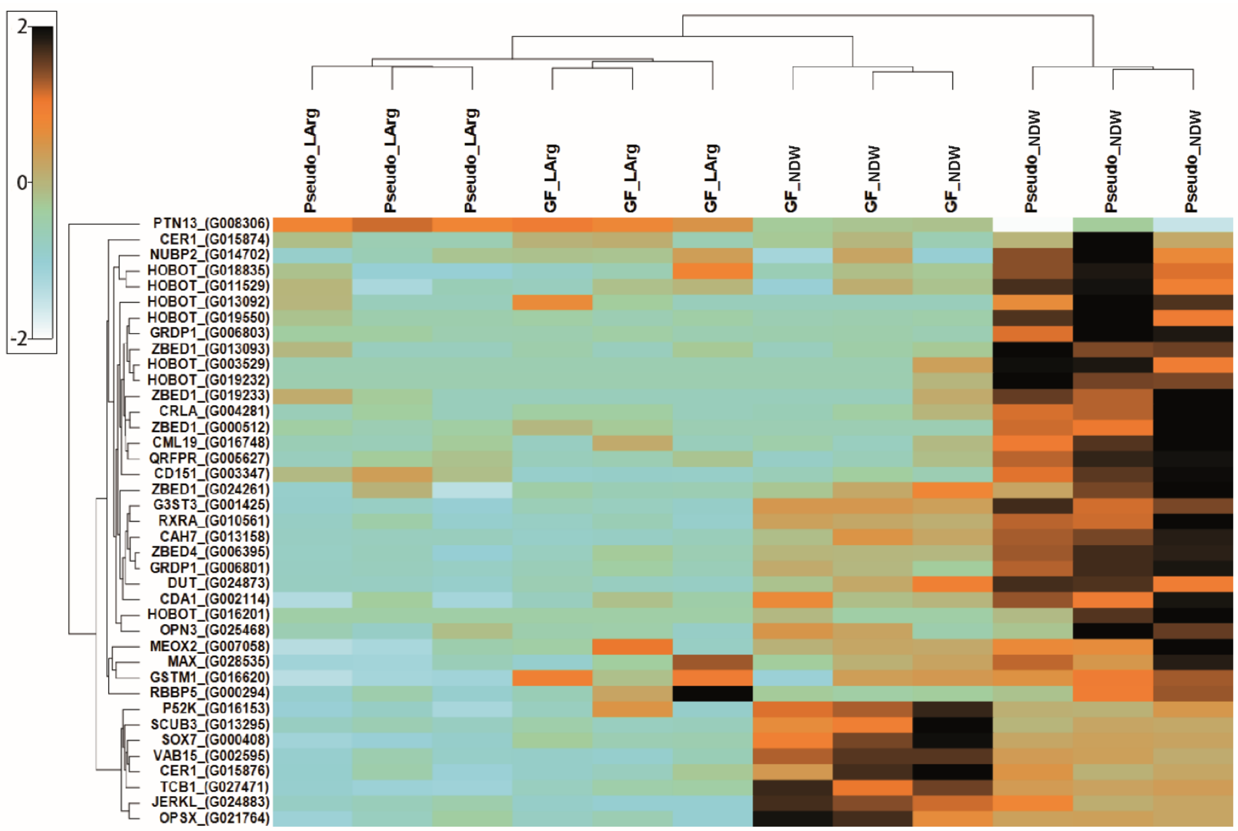

Fig. S14. Heatmap of all annotated genes that are differentially expressed (based on Z-score values) in *Hydra* polyps mono-colonized with *Pseudomonas alcaligenes* T3 (Pseudo) exposed to nutrient-deficient water (NDW) compared to germ-free (GF) polyps in nutrient-deficient water (NDW).

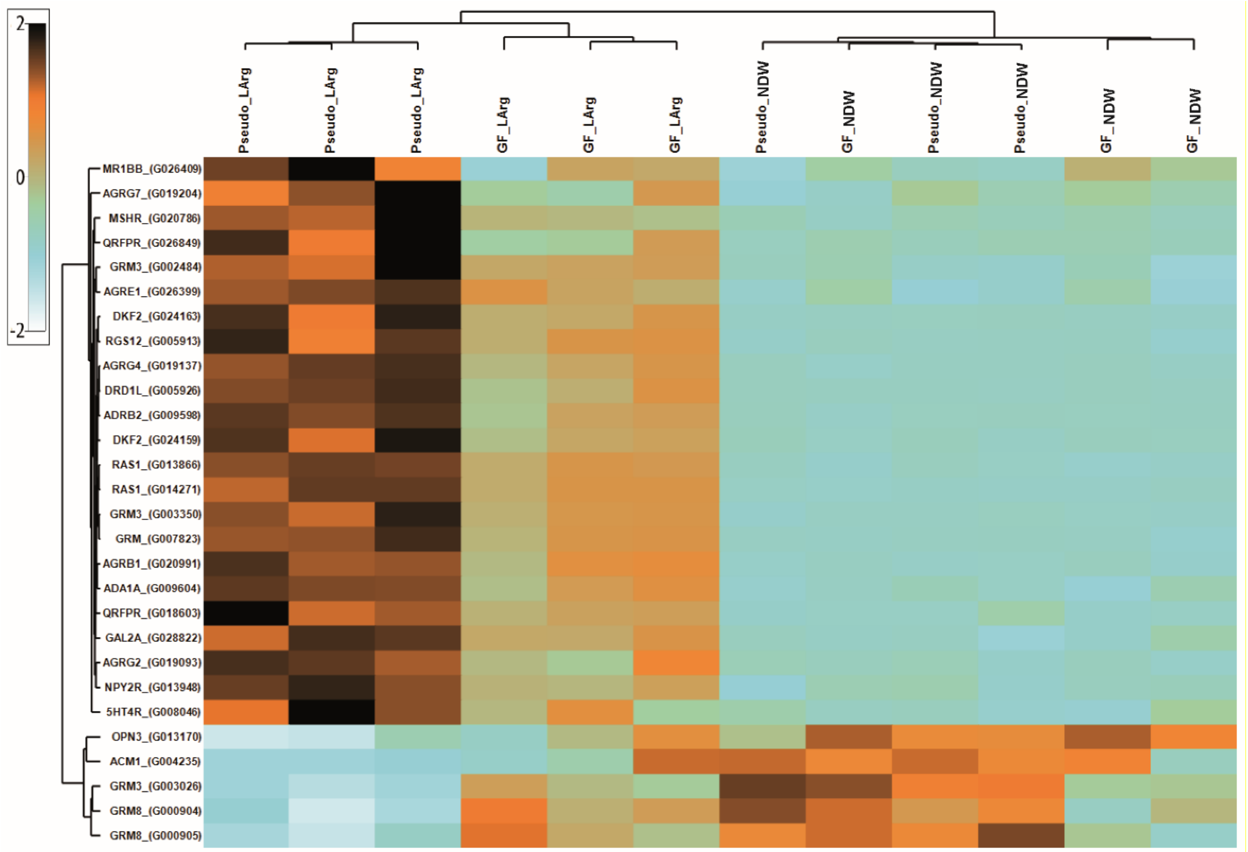

Fig. S15. Heatmap of differential expressed G-proteins (based on Z-score values) in *Hydra* polyps mono-colonized with *Pseudomonas alcaligenes* T3 (Pseudo) and exposed to L-arginine (LArg) compared to either mono-colonized polyps with *Pseudomonas alcaligenes* T3 (Pseudo) in nutrient-deficient water (NDW) or to germ-free (GF) polyps in L-arginine (LArg) or nutrient-deficient water (NDW).

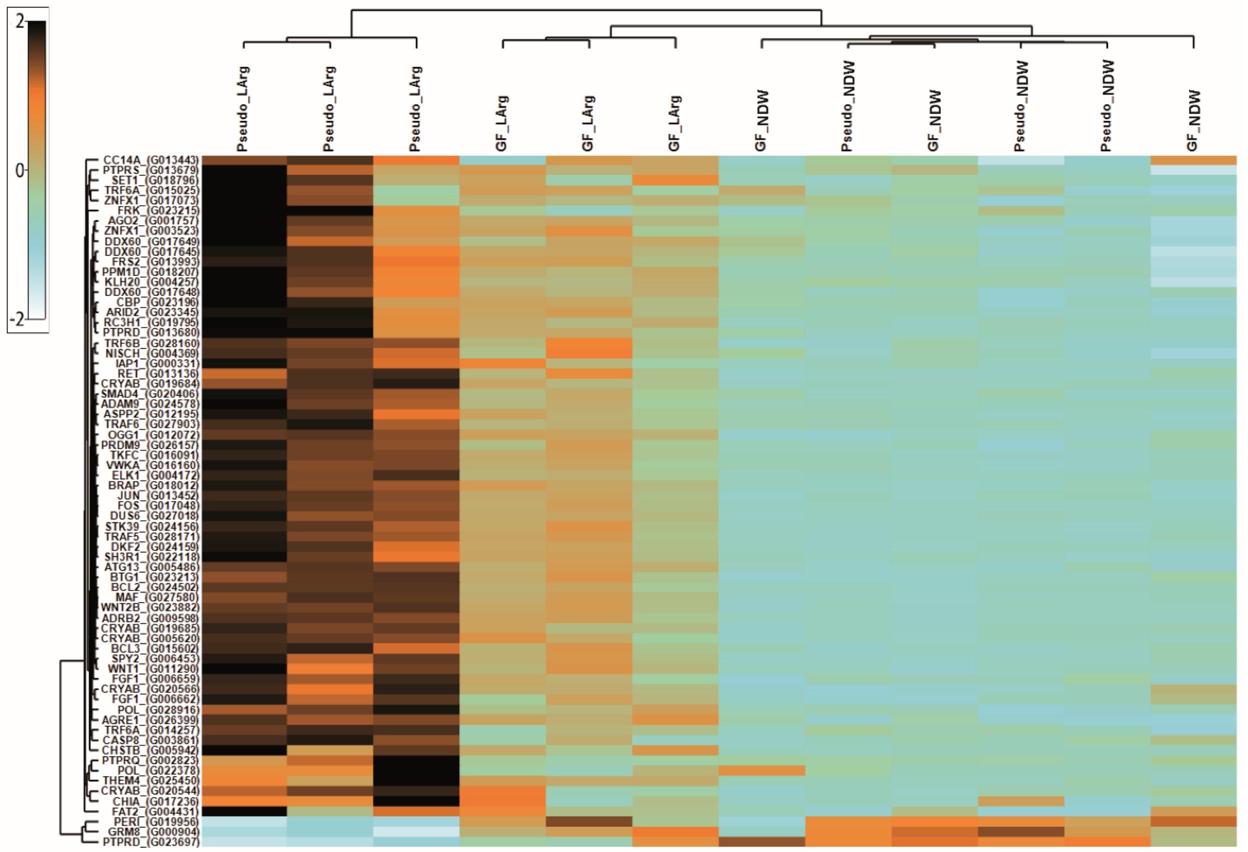

Fig. S16. Heatmap of differential expressed immune responsive genes (based on Z-score values) in *Hydra* polyps mono-colonized with *Pseudomonas alcaligenes* T3 (Pseudo) and exposed to L-arginine (LArg) compared to either mono-colonized polyps with *Pseudomonas alcaligenes* T3 (Pseudo) in nutrient-deficient water (NDW) or to germ-free (GF) polyps in L-arginine (LArg) or nutrient-deficient water (NDW).

Table S1. Nutrient load in mg/l of sterile (0.02 μm) filtered lake water.

	**DOC**	**Phosphate**	**Nitrogen**	**C/N**
Postsee	9.1	0.044	1.10	8.3
Lanker See	8.9	0.014	0.78	11.4
Tresdorfer See	10.0	0.011	2.00	5.0
Selenter See	6.4	0.005	0.57	11.2
Plußsee	6.9	0.017	0.89	7.8
Schluensee	0.1	0.006	0.46	0.2
